# Supplementary material for: Tissue-specific activation of gene expression by the Synergistic Activation Mediator (SAM) CRISPRa system in mice
Source: Nat Commun. 2021 May 13;12:2770. doi: 10.1038/s41467-021-22932-4 (PMC8119962; doi:10.1038/s41467-021-22932-4)
Supplement: Supplementary file 1 — Supplementary Information [file 41467_2021_22932_MOESM1_ESM.pdf]

Figure Supplementary 1:

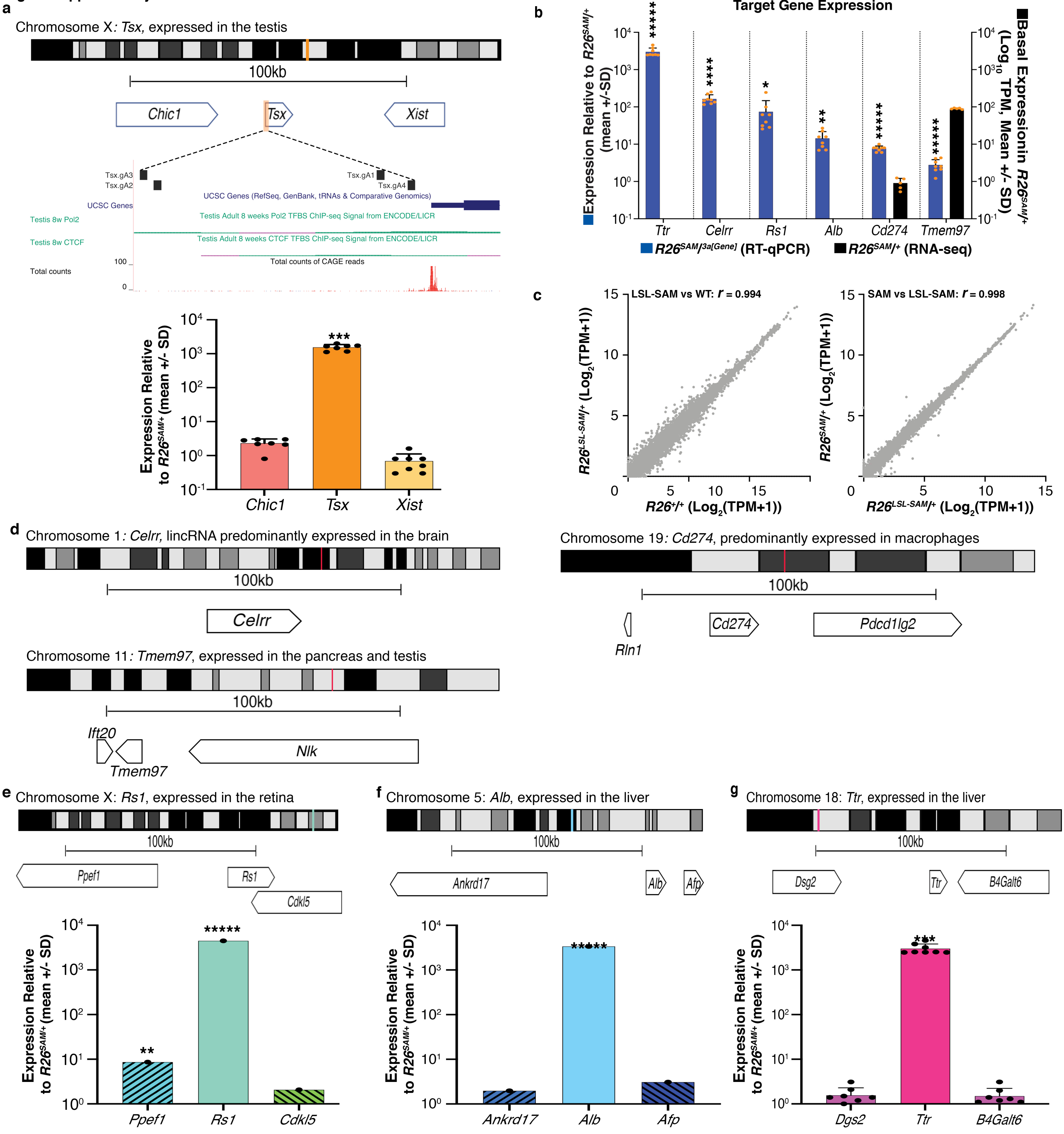

Figure Supplementary 1: Impact of *R26*^{SAM} on mESC genome (a) *R26*^{4aTsx} is comprised of two guides targeting the 300bp directly upstream of the TSS and another two guides targeting an upstream enhancer region (750bp upstream). RT-qPCR was completed on *R26*$^{SAM/4aTsx}$ targeted mESC with four technical reps and expression values relative to *R26*$^{SAM/+}$ were plotted as the mean per group +/- SD (p = 0.000308). **(b)** Expression values of target genes in mESC: Left axis, expression of target gene in *R26*$^{SAM/3a[Gene]}$ relative to *R26*$^{SAM/+}$ cells plotted as the mean per group plus or minus the standard deviation (+/- SD). Eight clones per target were assessed with four technical replicates each (*Ttr*: p = 0.000005; *Celrr*: p = 0.000014; *Rs1*: p = 0.048101; *Alb*: p = 0.001358; *Cd274*: p = 0.000001; *Tmem97*: p = 0.000001). Right axis, expression of target genes in *R26*$^{SAM/+}$ cells as determined by RNA-seq. The transcriptome of one clone was sequenced with five technical replicates to provide the number of target-specific transcripts per million (TPM) RNA molecules. **(c)** RNA-seq characterization of mESC. The transcriptome of each clone was sequenced with five technical replicates to determine the effect of protected or active expression of dCas9^{SAM} in mESC. Left, *R26*$^{LSL-SAM/+}$ compared to *R26*$^{+/+}$ (WT) (r = 0.998). Right, *R26*$^{SAM/+}$ compared to *R26*$^{LSL-SAM/+}$ (r = 0.994). **(d)** Genomic context of additional genes selected for upregulation by *R26* gRNA arrays. Genomic context and RT-qPCR of **(e)** *Rs1* (*Rs1*: p = 0.000001; *Ppef1*: p = 0.001542), **(f)** *Alb* (p = 0.000001), and **(g)** *Ttr* (p = 0.00014). Four technical reps were completed for each gene and expression values relative to *R26*$^{SAM/+}$ were plotted as the mean per group +/- SD. Statistics: Asterisks (*) indicates significance, and the number of asterisks (*) indicates the number of 0s after the decimal point. One-tailed, unpaired Student's *t*-test for **(a-b, e-g)**, Pearson's correlation for **(c)**.

Figure Supplementary 2

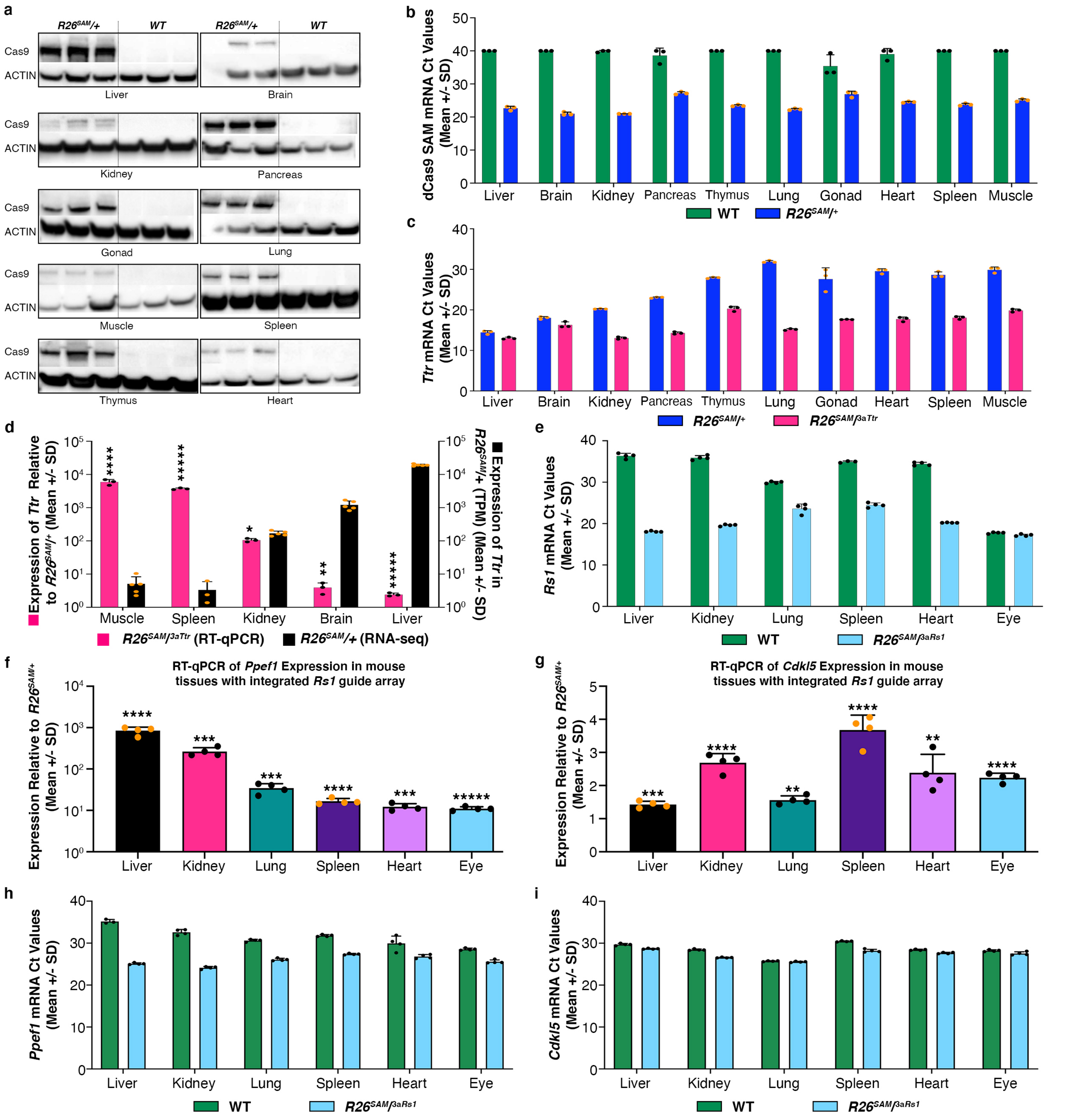

Figure Supplementary 2: *In vivo* characterization of the impact of $R26^{SAM}$ expression (a) Western blot showing dCas9^{SAM} (219 kDa) expression in tissues harboring $R26^{SAM/+}$. dCas9^{SAM} signals are normalized to actin (41 kDa). Independent replicates were not conducted. **(b)** Average cycle threshold (ct) values of dCas9^{SAM} expression per tissue plotted as the mean per group +/- SD (n = 3 mice per tissue). *B2m* ct values are plotted for reference. **(c)** Average ct values of *Ttr* expression per tissue plotted as the mean per group +/- SD (n = 3 mice per tissue). **(d)** Expression values of *Ttr* in tissues plotted as the mean per group +/- SD: Left axis, expression relative to R26$^{SAM/+}$ mouse tissues. *p*-values of each tissue can be found in Supplementary Table 8. Five mice per tissue were assessed with four technical reps each. Right axis, expression as determined by RNA-seq. The transcriptomes of five mouse tissues from five mice were sequenced to determine the number of target specific TPM. **(e)** Average ct values of *Rs1* in $R26^{SAM/3aRs1}$ mouse tissues plotted as the mean per group +/- SD (n = 4 mice per tissue). *Rs1* neighboring gene expression was evaluated for **(f)** *Ppef1* and **(g)** *Cdkl5* in six tissues from $R26^{SAM/3aRs1}$ mice by RT-qPCR (n = 4) and plotted as the mean per group +/- SD. *p*-values of each tissue can be found in Supplementary Table 8. Average ct values of **(h)** *Ppef1* and **(i)** *Cdkl5* in $R26^{SAM/3aRs1}$ mice and plotted as the mean per group +/- SD (n = 4). Statistics: Asterisks (*) indicates significance, and the number of asterisks (*) indicates the number of 0s after the decimal point. One-tailed, unpaired Student's *t*-test for **(d,f,g)**.

Figure Supplementary 3

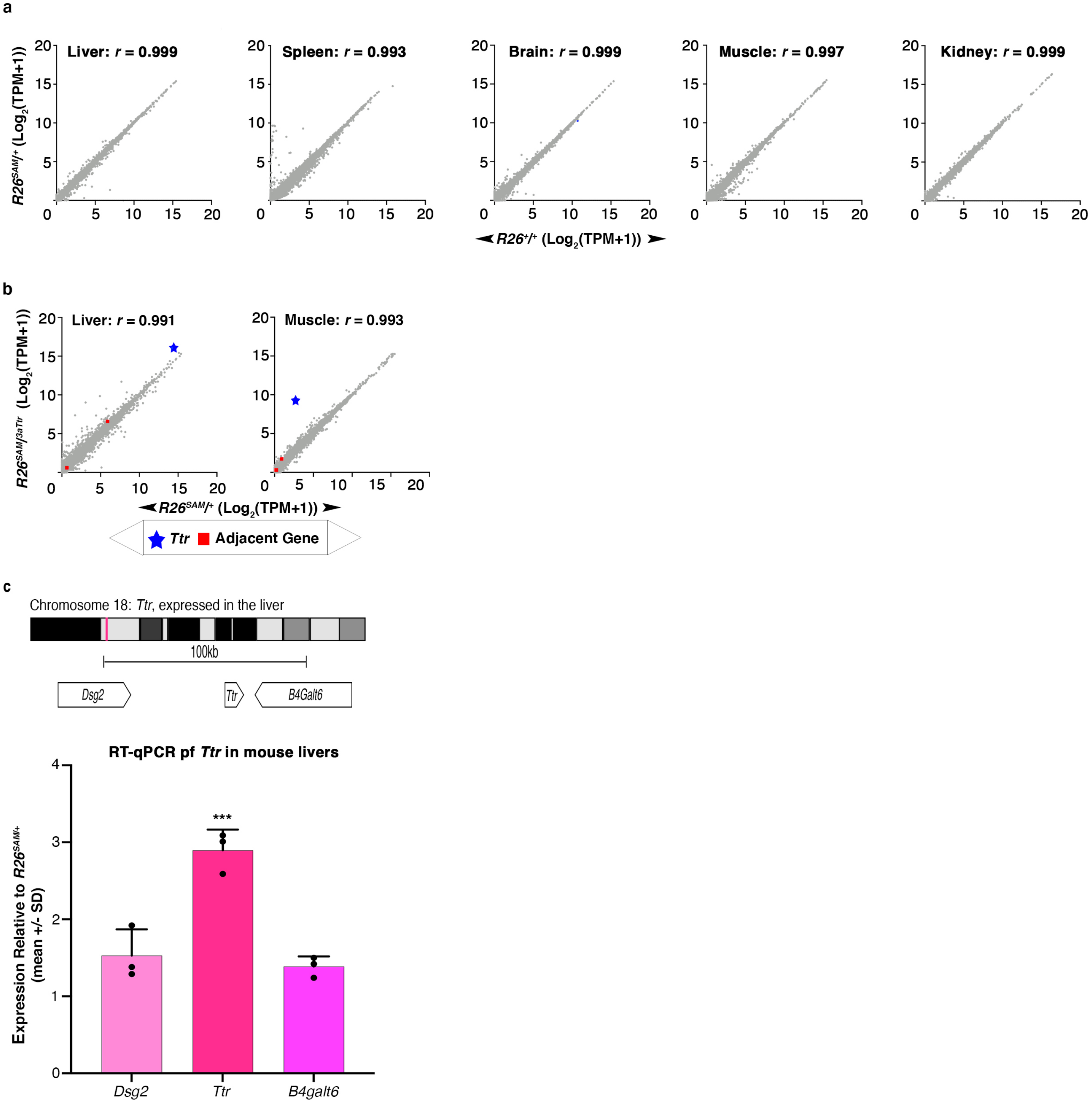

Figure Supplementary 3: RNA-seq characterization of the impact of *in vivo R26*^{SAM} expression (a) RNA-seq characterization of *R26*$^{SAM/+}$ mouse tissues. *p*-values of each tissue can be found in Supplementary Table 9. The transcriptome of each tissue from five mice was sequenced with five technical replicates to determine the effect of protected or active expression of dCas9^{SAM} compared to each respective parental line. **(b)** RNA-seq characterization of *R26*$^{SAM/3aTtr}$ mouse tissues versus the parental *R26*$^{SAM/+}$ line. The target gene is noted with a blue star and the adjacent genes with red squares (n = 5 mice per tissue). Liver: r = 0.991; Muscle: r = 0.993 **(c)** RT-qPCR analysis of the effect of SAM activation on *Ttr* neighboring genes in mice plotted as the mean per group +/- SD (n = 3 mouse livers) (p = 0.00014). Statistics: Asterisks (*) indicates significance, and the number of asterisks (*) indicates the number of 0s after the decimal point. One-tailed, unpaired Student's *t*-test for **(c)**, Pearson's correlation **(a,b)**.

Figure Supplementary 4

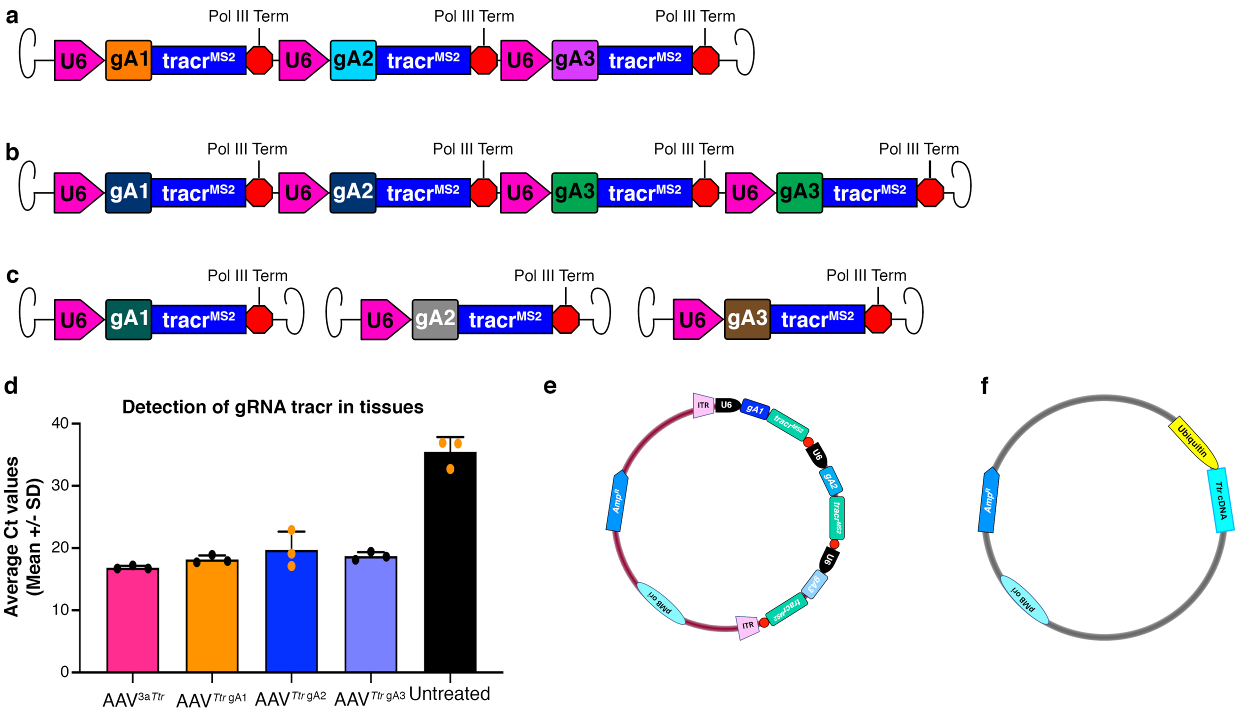

Figure Supplementary 4: Constructs (a) Generalized schematic of AAV guide arrays expressing 3 activating guides. Each guide is driven by a U6 promoter and separated by an extended Pol III termination sequence. **(b)** Generalized schematic of AAV guide arrays expressing four activating guides. **(c)** Generalized schematic of AAV single guide expression constructs. **(d)** RT-qPCR evaluation of tracr in liver samples post treatment with AAV delivered single guides or arrays. Ct values for assays were plotted as mean Ct value +/- SD. The array is expected to have three tracr copies while each single guide has one. **(e)** Schematic of the unpackaged 3aTtr plasmid. **(f)** Schematic of the HDD plasmid expressing *Ttr* cDNA from the *Ubiquitin* promoter.

Figure Supplementary 5

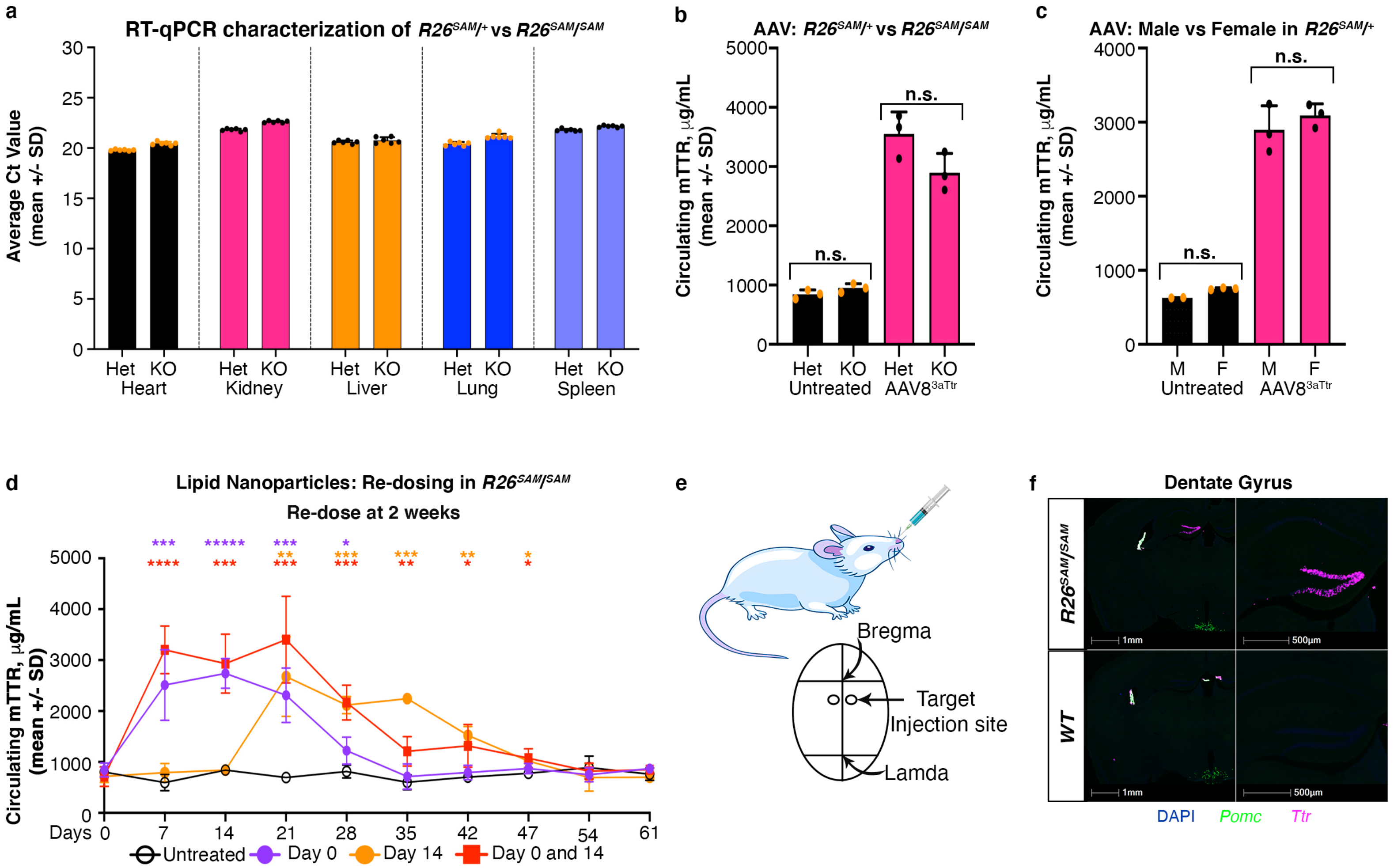

Figure Supplementary 5: SAM Guide Delivery Approaches (a) Average RT-qPCR Ct values of dCas9^{SAM} expression in selected organs plotted as the mean per group +/- SD (n = 6). (b) AAV8^{3aTtr} was delivered to *R26*$^{SAM/+}$ heterozygous (n = 3) and homozygous (n = 3) mice by tail vein injection and ELISA was employed to determine protein levels in each mouse. Values were plotted as the mean per group +/- SD. **(c)** AAV8^{3aTtr} was delivered to *R26*$^{SAM/+}$ male (n = 3) and female (n = 6) mice by tail vein injection. Circulating mTTR levels were determined for each mouse by ELISA. Serum values were plotted as the mean per group +/- SD. **(d)** LNP particles were formulated with 0.5mpk of synthetic *Ttr* gA2 SAM guides and introduced to *R26*$^{SAM/SAM}$ mice (n = 5) at zero and/or two weeks. Protein expression levels were determined by ELISA with weekly bleeds and all values are plotted as mean +/- SD. Sample *p*-values can be found in Supplementary Table 11. **(e)** Stereotactic approach to inject AAV8^{3aTtr} to the arcuate nucleus of the hypothalamus. **(f)** RNAscope analysis of *Ttr* expression in the dentate gyrus of *R26*$^{SAM/SAM}$ mouse (n = 1) DAPI (blue), *Pomc* (green) and *Ttr* (magenta). Statistics: Asterisks (*) indicates significance, and the number of asterisks (*) indicates the number of 0s after the decimal point. One-tailed, unpaired Student's t-test for **(b-d)**.

Figure Supplementary 6

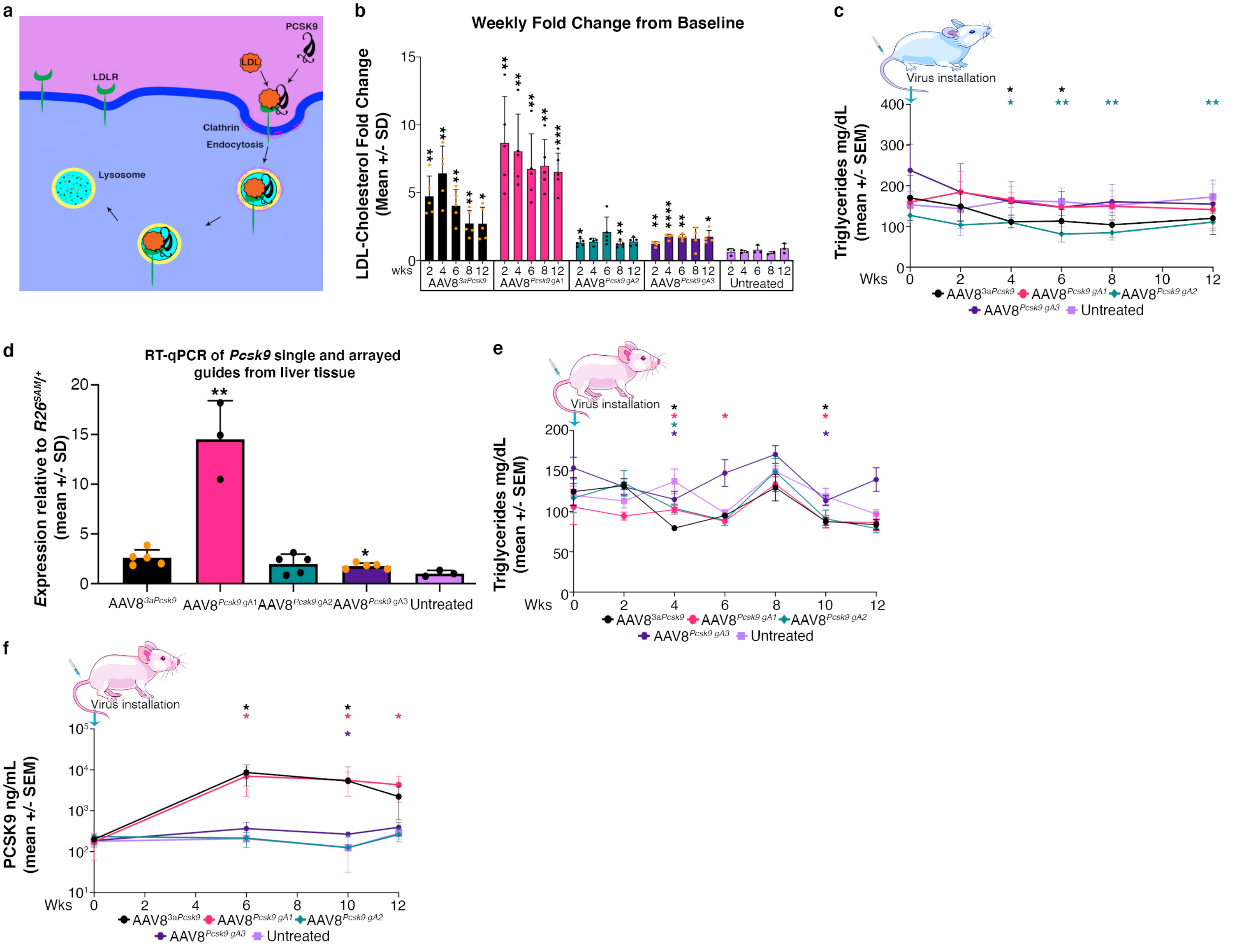

Figure Supplementary 6: Upregulation of *Pcsk9* and its effect on lipid metabolism (a) PCSK9 reduces LDLR levels on the plasma membrane. **(b)** LDL levels were plotted as mean fold change +/- SD per group relative to baseline LDL-Cholesterol levels (n = 5). Sample *p*-values can be found in Supplementary Table 12. **(c)** Triglyceride levels from male mouse study were plotted as mean value +/- SD (n = 5). Sample *p*-values can be found in Supplementary Table 12. **(d)** Expression of *Pcsk9* in the liver relative to untreated $R26^{SAM/+}$. (Guide gA1: $p = 0.003808$; Guide gA3: $p = 0.015013$). All values are plotted as mean per group +/- SEM (n = 5) **(e)** Triglyceride levels from the female mouse study were plotted as mean value +/- SEM (n = 5) Sample *p*-values can be found in Supplementary Table 12. **(f)** Serum PCSK9 levels from the female mouse study were plotted as mean value +/- SEM (n = 5). Sample *p*-values can be found in Supplementary Table 12. Statistics: Asterisks (*) indicates significance, and the number of asterisks (*) indicates the number of 0s after the decimal point. One-tailed, unpaired Student's *t*-test for **(b-f)**.

Figure Supplementary 7

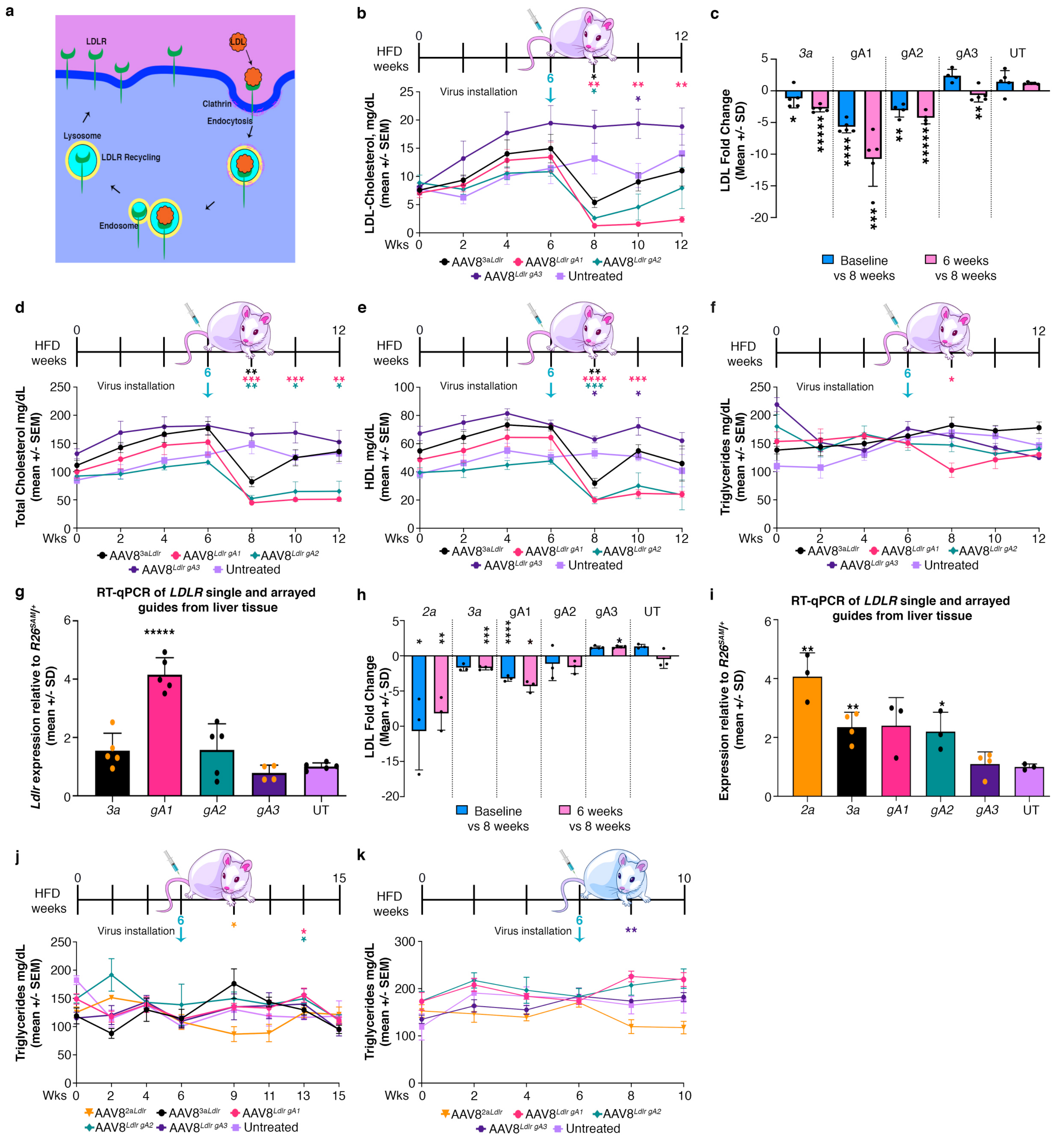

Figure Supplementary 7: Increased expression of *Ldlr* reduces total cholesterol levels (a) LDLR internalizes circulating LDL through endocytosis. **(b)** Homozygous female $R26^{SAM}$ study mice were placed on a HFD for six weeks prior to AAV8^{Ldlr} activating viruses being introduced (n = 4). LDL-Cholesterol levels were plotted as mean +/- SEM per group. Sample *p*-values can be found in Supplementary Table 13. **(c)** LDL levels were plotted as mean fold change +/- SD per group from baseline to eight weeks (two weeks post injection, orange) and from six weeks to eight weeks (blue) (n = 5). Sample *p*-values can be found in Supplementary Table 13. **(d)** Total cholesterol (n = 5), **(e)** HDL (n = 5), and **(f)** Triglyceride levels (n = 5) were plotted as mean value +/- SEM. Sample *p*-values can be found in Supplementary Table 13. **(g)** Relative expression of *Ldlr* in the liver relative to $R26^{SAM/+}$ (p = 0.000002). All values are plotted as mean per group +/- SD (n = 5). **(h)** LDL levels were plotted as mean fold change +/- SD per group from baseline to eight weeks (two weeks post injection, orange) and from six weeks to eight weeks (blue) (n = 3). Sample *p*-values can be found in Supplementary Table 13. **(i)** Relative expression of *Ldlr* in the liver relative to $R26^{SAM/+}$. All values are plotted as mean per group +/- SD (n = 5). **(j)** Triglyceride levels from the female study mice were plotted as mean value +/- SD (n = 3). Sample *p*-values can be found in Supplementary Table 13. **(k)** Triglyceride levels from male study mice were plotted as mean value +/- SD (n = 3). Guide gA3, 8 weeks: p = 0.005016. Statistics: Asterisks (*) indicates significance, and the number of asterisks (*) indicates the number of 0s after the decimal point. One-tailed, unpaired Student's *t*-test for **(b-k)**.

Supplementary Figure 8: MacVector sequence report from AAV8^{3aTtr} sanger sequence alignment. Positions of mismatches, if any, are displayed in the green highlight. Sanger reads are displayed as blue arrows.

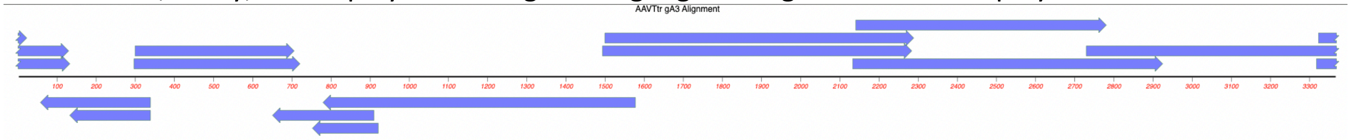

Potential sequencing problems report for AAV3aTtr Alignment										
This tab is primarily designed to help you identify sequencing errors with genomic sequences and NGS data.										
Once you have a final sequence, run Align To Reference with it using a representative sample of your data.										
We recommend no more than 5 million reads unless you have more than 16 GB RAM. When you align, make sure										
you set the Hash Value to 12 and Sensitivity to 3 or less for optimal performance.										
Ref Base is the residue in the reference sequence										
Cons Base is the residue in the consensus sequence										
A, G, C and T are the counts of the occurence of each residue in reads that cross the location										
Gaps are the number of reads with gaps at that location										
Ambig are the number of reads with ambiguities at that location										
Clip'd are the number of reads that are clipped (greyed out) at that location										
Score is calculated by adding 2 points for each mismatch and 1 for each gap/ambiguity/clipped read										
Only the highest-scoring (i.e. 'worst') 2,000 positions are displayed in the table										
Pos.	Ref Base	Cons Base	Score	A	C	G	T	Gaps	Ambig	Clip'd
Mean coverage = 1.4, Standard Deviation = 0.5										
The following regions have NO coverage:										
From	To									
71	72									
1705										

Supplementary Figure 9: MacVector sequence report from AAV8$^{Ttr\text{-}gA1}$ sanger sequence alignment. Positions of mismatches, if any, are displayed in the green highlight. Sanger reads are displayed as blue arrows.

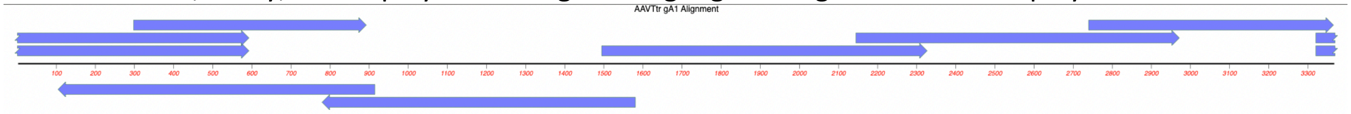

Potential sequencing problems report for AAVTtr gA1 Alignment										
This tab is primarily designed to help you identify sequencing errors with genomic sequences and NGS data.										
Once you have a final sequence, run Align To Reference with it using a representative sample of your data.										
We recommend no more than 5 million reads unless you have more than 16 GB RAM. When you align, make sure										
you set the Hash Value to 12 and Sensitivity to 3 or less for optimal performance.										
Ref Base is the residue in the reference sequence										
Cons Base is the residue in the consensus sequence										
A, G, C and T are the counts of the occurence of each residue in reads that cross the location										
Gaps are the number of reads with gaps at that location										
Ambig are the number of reads with ambiguities at that location										
Clip'd are the number of reads that are clipped (greyed out) at that location										
Score is calculated by adding 2 points for each mismatch and 1 for each gap/ambiguity/clipped read										
Only the highest-scoring (i.e. 'worst') 2,000 positions are displayed in the table										
Pos.	Ref Base	Cons Base	Score	A	C	G	T	Gaps	Ambig	Clip'd
Mean coverage = 1.7, Standard Deviation = 1.0										
There are no regions with no coverage.										

Supplementary Figure 10: MacVector sequence report from AAV8$^{Ttr\text{-}gA2}$ sanger sequence alignment. Positions of mismatches, if any, are displayed in the green highlight. Sanger reads are displayed as blue arrows.

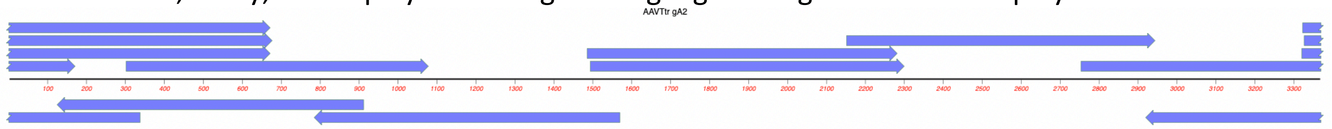

Potential sequencing problems report for AAVTtr gA2 Alignment										
This tab is primarily designed to help you identify sequencing errors with genomic sequences and NGS data.										
Once you have a final sequence, run Align To Reference with it using a representative sample of your data.										
We recommend no more than 5 million reads unless you have more than 16 GB RAM. When you align, make sure										
you set the Hash Value to 12 and Sensitivity to 3 or less for optimal performance.										
Ref Base is the residue in the reference sequence										
Cons Base is the residue in the consensus sequence										
A, G, C and T are the counts of the occurence of each residue in reads that cross the location										
Gaps are the number of reads with gaps at that location										
Ambig are the number of reads with ambiguities at that location										
Clip'd are the number of reads that are clipped (greyed out) at that location										
Score is calculated by adding 2 points for each mismatch and 1 for each gap/ambiguity/clipped read										
Only the highest-scoring (i.e. 'worst') 2,000 positions are displayed in the table										
Pos.	Ref Base	Cons Base	Score	A	C	G	T	Gaps	Ambig	Clip'd
Mean coverage = 2.5, Standard Deviation = 1.5										
There are no regions with no coverage.										

Supplementary Figure 11: MacVector sequence report from AAV8$^{Ttr\text{-}gA3}$ sanger sequence alignment. Positions of mismatches, if any, are displayed in the green highlight. Sanger reads are displayed as blue arrows.

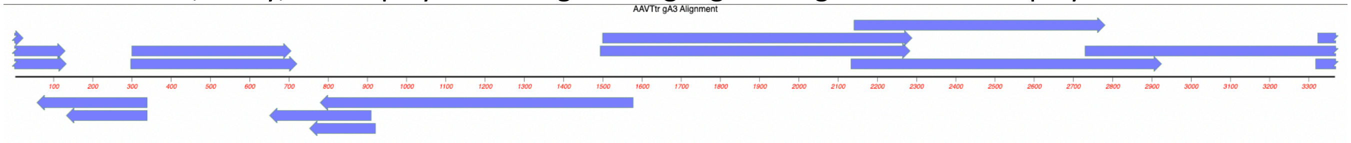

Potential sequencing problems report for AAVTtr gA3 Alignment										
This tab is primarily designed to help you identify sequencing errors with genomic sequences and NGS data.										
Once you have a final sequence, run Align To Reference with it using a representative sample of your data.										
We recommend no more than 5 million reads unless you have more than 16 GB RAM. When you align, make sure										
you set the Hash Value to 12 and Sensitivity to 3 or less for optimal performance.										
Ref Base is the residue in the reference sequence										
Cons Base is the residue in the consensus sequence										
A, G, C and T are the counts of the occurence of each residue in reads that cross the location										
Gaps are the number of reads with gaps at that location										
Ambig are the number of reads with ambiguities at that location										
Clip'd are the number of reads that are clipped (greyed out) at that location										
Score is calculated by adding 2 points for each mismatch and 1 for each gap/ambiguity/clipped read										
Only the highest-scoring (i.e. 'worst') 2,000 positions are displayed in the table										
Pos.	Ref Base	Cons Base	Score	A	C	G	T	Gaps	Ambig	Clip'd
Mean coverage = 1.9, Standard Deviation = 0.8										
There are no regions with no coverage.										

Supplementary Figure 12: MacVector sequence report from AAV8^{3aRs1} sanger sequence alignment. Positions of mismatches, if any, are displayed in the green highlight. Sanger reads are displayed as blue arrows.

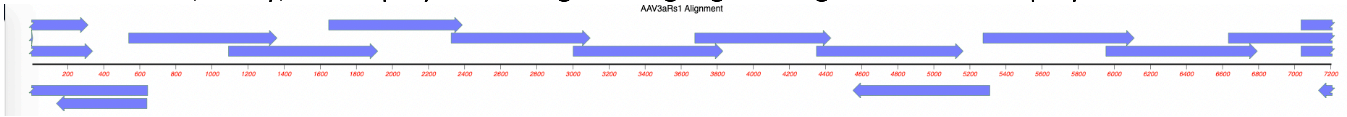

Potential sequencing problems report for AAV3aRs1										
This tab is primarily designed to help you identify sequencing errors with genomic sequences and NGS data.										
Once you have a final sequence, run Align To Reference with it using a representative sample of your data.										
We recommend no more than 5 million reads unless you have more than 16 GB RAM. When you align, make sure										
you set the Hash Value to 12 and Sensitivity to 3 or less for optimal performance.										
Ref Base is the residue in the reference sequence										
Cons Base is the residue in the consensus sequence										
A, G, C and T are the counts of the occurence of each residue in reads that cross the location										
Gaps are the number of reads with gaps at that location										
Ambig are the number of reads with ambiguities at that location										
Clip'd are the number of reads that are clipped (greyed out) at that location										
Score is calculated by adding 2 points for each mismatch and 1 for each gap/ambiguity/clipped read										
Only the highest-scoring (i.e. 'worst') 2,000 positions are displayed in the table										
Pos.	Ref Base	Cons Base	Score	A	C	G	T	Gaps	Ambig	Clip'd
Mean coverage = 1.5, Standard Deviation = 0.7										
There are no regions with no coverage.										

Supplementary Figure 13: MacVector sequence report from AAV8$^{Rs1\text{-gA1}}$ sanger sequence alignment. Positions of mismatches, if any, are displayed in the green highlight. Sanger reads are displayed as blue arrows.

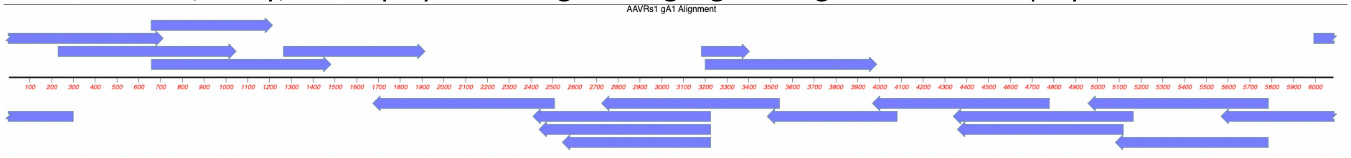

Potential sequencing problems report for AAVRs1 gA1 Alignment										
This tab is primarily designed to help you identify sequencing errors with genomic sequences and NGS data.										
Once you have a final sequence, run Align To Reference with it using a representative sample of your data.										
We recommend no more than 5 million reads unless you have more than 16 GB RAM. When you align, make sure										
you set the Hash Value to 12 and Sensitivity to 3 or less for optimal performance.										
Ref Base is the residue in the reference sequence										
Cons Base is the residue in the consensus sequence										
A, G, C and T are the counts of the occurence of each residue in reads that cross the location										
Gaps are the number of reads with gaps at that location										
Ambig are the number of reads with ambiguities at that location										
Clip'd are the number of reads that are clipped (greyed out) at that location										
Score is calculated by adding 2 points for each mismatch and 1 for each gap/ambiguity/clipped read										
Only the highest-scoring (i.e. 'worst') 2,000 positions are displayed in the table										
Pos.	Ref Base	Cons Base	Score	A	C	G	T	Gaps	Ambig	Clip'd
Mean coverage = 2.3, Standard Deviation = 0.9										
There are no regions with no coverage.										

Supplementary Figure 14: MacVector sequence report from $AAV8^{\mathit{Rs1}\text{-}gA2}$ sanger sequence alignment. Positions of mismatches, if any, are displayed in the green highlight. Sanger reads are displayed as blue arrows.

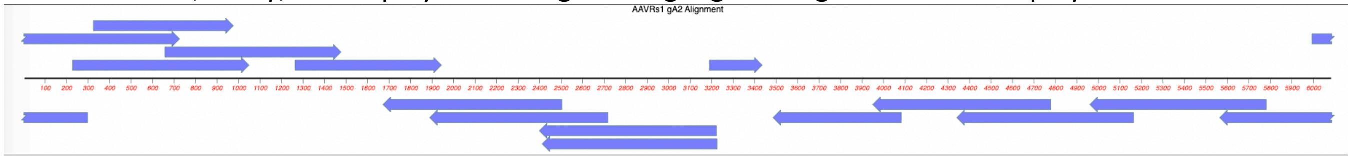

Potential sequencing problems report for AAVRs1 gA2 Alignment										
This tab is primarily designed to help you identify sequencing errors with genomic sequences and NGS data.										
Once you have a final sequence, run Align To Reference with it using a representative sample of your data.										
We recommend no more than 5 million reads unless you have more than 16 GB RAM. When you align, make sure										
you set the Hash Value to 12 and Sensitivity to 3 or less for optimal performance.										
Ref Base is the residue in the reference sequence										
Cons Base is the residue in the consensus sequence										
A, G, C and T are the counts of the occurence of each residue in reads that cross the location										
Gaps are the number of reads with gaps at that location										
Ambig are the number of reads with ambiguities at that location										
Clip'd are the number of reads that are clipped (greyed out) at that location										
Score is calculated by adding 2 points for each mismatch and 1 for each gap/ambiguity/clipped read										
Only the highest-scoring (i.e. 'worst') 2,000 positions are displayed in the table										
Pos.	Ref Base	Cons Base	Score	A	C	G	T	Gaps	Ambig	Clip'd
Mean coverage = 1.9, Standard Deviation = 0.8										
The following regions have NO coverage:										
From	To									
3435	3485									

Supplementary Figure 15: MacVector sequence report from AAV8$^{Rs1\text{-}gA3}$ sanger sequence alignment. Positions of mismatches, if any, are displayed in the green highlight. Sanger reads are displayed as blue arrows.

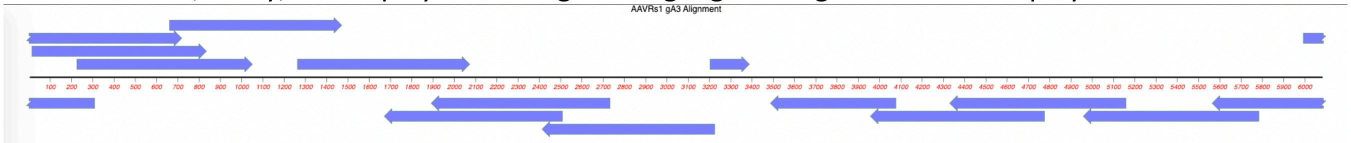

Potential sequencing problems report for AAVRs1 gA3 Alignment										
This tab is primarily designed to help you identify sequencing errors with genomic sequences and NGS data.										
Once you have a final sequence, run Align To Reference with it using a representative sample of your data.										
We recommend no more than 5 million reads unless you have more than 16 GB RAM. When you align, make sure										
you set the Hash Value to 12 and Sensitivity to 3 or less for optimal performance.										
Ref Base is the residue in the reference sequence										
Cons Base is the residue in the consensus sequence										
A, G, C and T are the counts of the occurence of each residue in reads that cross the location										
Gaps are the number of reads with gaps at that location										
Ambig are the number of reads with ambiguities at that location										
Clip'd are the number of reads that are clipped (greyed out) at that location										
Score is calculated by adding 2 points for each mismatch and 1 for each gap/ambiguity/clipped read										
Only the highest-scoring (i.e. 'worst') 2,000 positions are displayed in the table										
Pos.	Ref Base	Cons Base	Score	A	C	G	T	Gaps	Ambig	Clip'd
Mean coverage = 1.8, Standard Deviation = 0.8										
The following regions have NO coverage:										
From	To									
3387	3486									

Supplementary Figure 16: MacVector sequence report from AAV8^{4aTsx} sanger sequence alignment. Positions of mismatches, if any, are displayed in the green highlight. Sanger reads are displayed as blue arrows.

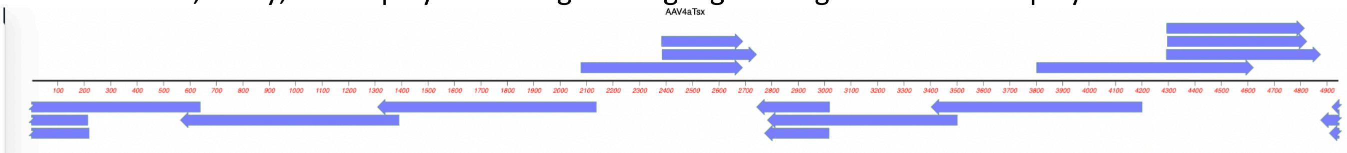

Potential sequencing problems report for AAV4aTsx										
This tab is primarily designed to help you identify sequencing errors with genomic sequences and NGS data.										
Once you have a final sequence, run Align To Reference with it using a representative sample of your data.										
We recommend no more than 5 million reads unless you have more than 16 GB RAM. When you align, make sure										
you set the Hash Value to 12 and Sensitivity to 3 or less for optimal performance.										
Ref Base is the residue in the reference sequence										
Cons Base is the residue in the consensus sequence										
A, G, C and T are the counts of the occurence of each residue in reads that cross the location										
Gaps are the number of reads with gaps at that location										
Ambig are the number of reads with ambiguities at that location										
Clip'd are the number of reads that are clipped (greyed out) at that location										
Score is calculated by adding 2 points for each mismatch and 1 for each gap/ambiguity/clipped read										
Only the highest-scoring (i.e. 'worst') 2,000 positions are displayed in the table										
Pos.	Ref Base	Cons Base	Score	A	C	G	T	Gaps	Ambig	Clip'd
Mean coverage = 1.8, Standard Deviation = 0.9										
The following regions have NO coverage:										
From	To									
2199										

Supplementary Figure 17: MacVector sequence report from AAV8$^{Tsx\text{-}gA1}$ sanger sequence alignment. Positions of mismatches, if any, are displayed in the green highlight. Sanger reads are displayed as blue arrows.

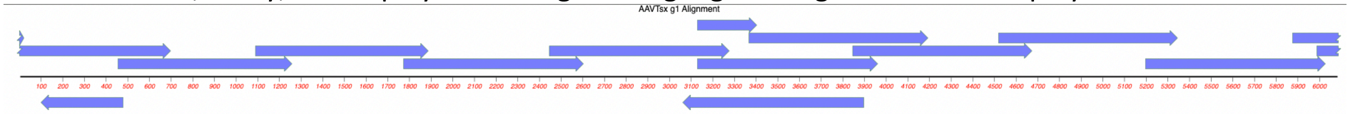

Potential sequencing problems report for AAVTsx g1 Alignment										
This tab is primarily designed to help you identify sequencing errors with genomic sequences and NGS data.										
Once you have a final sequence, run Align To Reference with it using a representative sample of your data.										
We recommend no more than 5 million reads unless you have more than 16 GB RAM. When you align, make sure										
you set the Hash Value to 12 and Sensitivity to 3 or less for optimal performance.										
Ref Base is the residue in the reference sequence										
Cons Base is the residue in the consensus sequence										
A, G, C and T are the counts of the occurence of each residue in reads that cross the location										
Gaps are the number of reads with gaps at that location										
Ambig are the number of reads with ambiguities at that location										
Clip'd are the number of reads that are clipped (greyed out) at that location										
Score is calculated by adding 2 points for each mismatch and 1 for each gap/ambiguity/clipped read										
Only the highest-scoring (i.e. 'worst') 2,000 positions are displayed in the table										
Pos.	Ref Base	Cons Base	Score	A	C	G	T	Gaps	Ambig	Clip'd
Mean coverage = 1.6, Standard Deviation = 0.8										
There are no regions with no coverage.										

Supplementary Figure 18: MacVector sequence report from AAV8$^{Tsx\text{-}gA2}$ sanger sequence alignment. Positions of mismatches, if any, are displayed in the green highlight. Sanger reads are displayed as blue arrows.

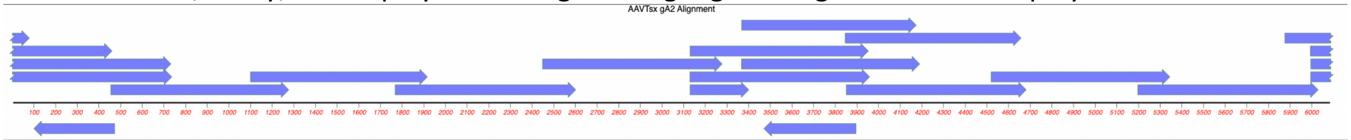

Potential sequencing problems report for AAVTsx gA2 Alignment										
This tab is primarily designed to help you identify sequencing errors with genomic sequences and NGS data.										
Once you have a final sequence, run Align To Reference with it using a representative sample of your data.										
We recommend no more than 5 million reads unless you have more than 16 GB RAM. When you align, make sure										
you set the Hash Value to 12 and Sensitivity to 3 or less for optimal performance.										
Ref Base is the residue in the reference sequence										
Cons Base is the residue in the consensus sequence										
A, G, C and T are the counts of the occurence of each residue in reads that cross the location										
Gaps are the number of reads with gaps at that location										
Ambig are the number of reads with ambiguities at that location										
Clip'd are the number of reads that are clipped (greyed out) at that location										
Score is calculated by adding 2 points for each mismatch and 1 for each gap/ambiguity/clipped read										
Only the highest-scoring (i.e. 'worst') 2,000 positions are displayed in the table										
Pos.	Ref Base	Cons Base	Score	A	C	G	T	Gaps	Ambig	Clip'd
Mean coverage = 2.2, Standard Deviation = 1.5										
There are no regions with no coverage.										

Supplementary Figure 19: MacVector sequence report from AAV8$^{\textit{Tsx}\text{-gA3}}$ sanger sequence alignment. Positions of mismatches, if any, are displayed in the green highlight. Sanger reads are displayed as blue arrows.

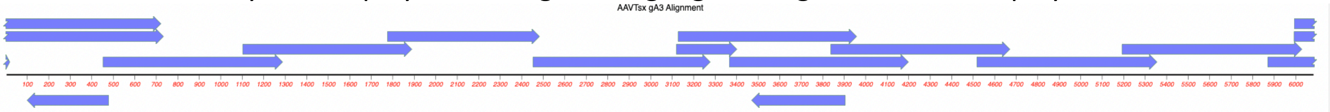

Potential sequencing problems report for AAVTsx gA3 Alignment										
This tab is primarily designed to help you identify sequencing errors with genomic sequences and NGS data.										
Once you have a final sequence, run Align To Reference with it using a representative sample of your data.										
We recommend no more than 5 million reads unless you have more than 16 GB RAM. When you align, make sure										
you set the Hash Value to 12 and Sensitivity to 3 or less for optimal performance.										
Ref Base is the residue in the reference sequence										
Cons Base is the residue in the consensus sequence										
A, G, C and T are the counts of the occurence of each residue in reads that cross the location										
Gaps are the number of reads with gaps at that location										
Ambig are the number of reads with ambiguities at that location										
Clip'd are the number of reads that are clipped (greyed out) at that location										
Score is calculated by adding 2 points for each mismatch and 1 for each gap/ambiguity/clipped read										
Only the highest-scoring (i.e. 'worst') 2,000 positions are displayed in the table										
Pos.	Ref Base	Cons Base	Score	A	C	G	T	Gaps	Ambig	Clip'd
Mean coverage = 1.7, Standard Deviation = 0.9										
There are no regions with no coverage.										

Supplementary Figure 20: MacVector sequence report from AAV8$^{Tsx\text{-}gA4}$ sanger sequence alignment. Positions of mismatches, if any, are displayed in the green highlight. Sanger reads are displayed as blue arrows.

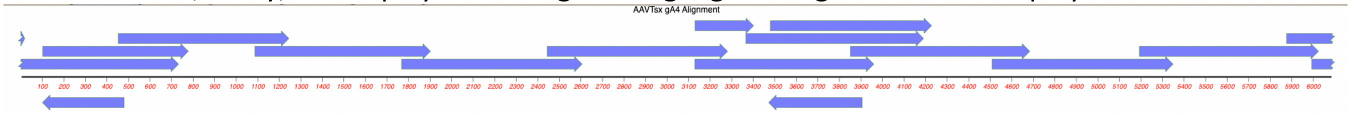

Potential sequencing problems report for AAV4aTsx										
This tab is primarily designed to help you identify sequencing errors with genomic sequences and NGS data.										
Once you have a final sequence, run Align To Reference with it using a representative sample of your data.										
We recommend no more than 5 million reads unless you have more than 16 GB RAM. When you align, make sure										
you set the Hash Value to 12 and Sensitivity to 3 or less for optimal performance.										
Ref Base is the residue in the reference sequence										
Cons Base is the residue in the consensus sequence										
A, G, C and T are the counts of the occurence of each residue in reads that cross the location										
Gaps are the number of reads with gaps at that location										
Ambig are the number of reads with ambiguities at that location										
Clip'd are the number of reads that are clipped (greyed out) at that location										
Score is calculated by adding 2 points for each mismatch and 1 for each gap/ambiguity/clipped read										
Only the highest-scoring (i.e. 'worst') 2,000 positions are displayed in the table										
Pos.	Ref Base	Cons Base	Score	A	C	G	T	Gaps	Ambig	Clip'd
Mean coverage = 1.8, Standard Deviation = 0.9										
The following regions have NO coverage:										
From	To									
2199										

Supplementary Figure 21: MacVector sequence report from AAV8$^{2aNgn3\text{-}2aBtc}$ sanger sequence alignment. Positions of mismatches, if any, are displayed in the green highlight. Sanger reads are displayed as blue arrows.

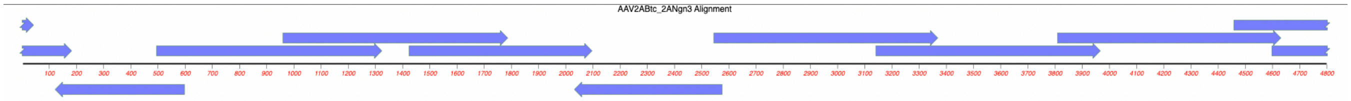

Potential sequencing problems report for AAV2ABtc_2ANgn3 Alignment

This tab is primarily designed to help you identify sequencing errors with genomic sequences and NGS data.
Once you have a final sequence, run Align To Reference with it using a representative sample of your data.
We recommend no more than 5 million reads unless you have more than 16 GB RAM. When you align, make sure
you set the Hash Value to 12 and Sensitivity to 3 or less for optimal performance.

Ref Base is the residue in the reference sequence
Cons Base is the residue in the consensus sequence
A, G, C and T are the counts of the occurence of each residue in reads that cross the location
Gaps are the number of reads with gaps at that location
Ambig are the number of reads with ambiguities at that location
Clip'd are the number of reads that are clipped (greyed out) at that location
Score is calculated by adding 2 points for each mismatch and 1 for each gap/ambiguity/clipped read
Only the highest-scoring (i.e. 'worst') 2,000 positions are displayed in the table

Pos.	Ref Base	Cons Base	Score	A	C	G	T	Gaps	Ambig	Clip'd

Mean coverage = 1.4, Standard Deviation = 0.5

There are no regions with no coverage.

There are no regions with coverage less than two standard deviations from the mean (1):

The following regions have coverage greater than two standard deviations from the mean (2).

From	To
4598	4629

Supplementary Figure 22: MacVector sequence report from AAV8$^{Ldlr\text{-}gA1}$ sanger sequence alignment. Positions of mismatches, if any, are displayed in the green highlight. Sanger reads are displayed as blue arrows.

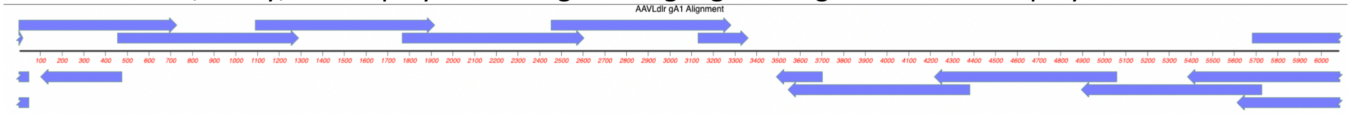

Potential sequencing problems report for AAVLdlr gA1 Alignment										
This tab is primarily designed to help you identify sequencing errors with genomic sequences and NGS data.										
Once you have a final sequence, run Align To Reference with it using a representative sample of your data.										
We recommend no more than 5 million reads unless you have more than 16 GB RAM. When you align, make sure										
you set the Hash Value to 12 and Sensitivity to 3 or less for optimal performance.										
Ref Base is the residue in the reference sequence										
Cons Base is the residue in the consensus sequence										
A, G, C and T are the counts of the occurence of each residue in reads that cross the location										
Gaps are the number of reads with gaps at that location										
Ambig are the number of reads with ambiguities at that location										
Clip'd are the number of reads that are clipped (greyed out) at that location										
Score is calculated by adding 2 points for each mismatch and 1 for each gap/ambiguity/clipped read										
Only the highest-scoring (i.e. 'worst') 2,000 positions are displayed in the table										
Pos.	Ref Base	Cons Base	Score	A	C	G	T	Gaps	Ambig	Clip'd
Mean coverage = 1.5, Standard Deviation = 0.7										
The following regions have NO coverage:										
From	To									
3360	3489									

Supplementary Figure 23: MacVector sequence report from AAV8$^{Ldlr\text{-}gA2}$ sanger sequence alignment. Positions of mismatches, if any, are displayed in the green highlight. Sanger reads are displayed as blue arrows.

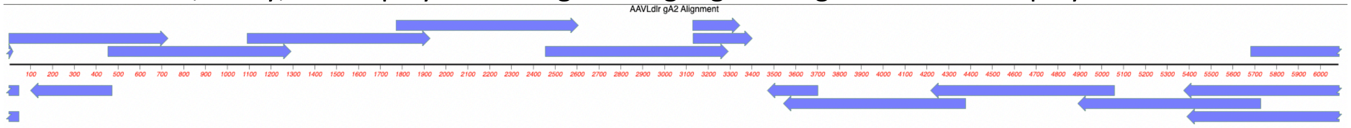

Potential sequencing problems report for AAVLdlr gA2 Alignment										
This tab is primarily designed to help you identify sequencing errors with genomic sequences and NGS data.										
Once you have a final sequence, run Align To Reference with it using a representative sample of your data.										
We recommend no more than 5 million reads unless you have more than 16 GB RAM. When you align, make sure										
you set the Hash Value to 12 and Sensitivity to 3 or less for optimal performance.										
Ref Base is the residue in the reference sequence										
Cons Base is the residue in the consensus sequence										
A, G, C and T are the counts of the occurence of each residue in reads that cross the location										
Gaps are the number of reads with gaps at that location										
Ambig are the number of reads with ambiguities at that location										
Clip'd are the number of reads that are clipped (greyed out) at that location										
Score is calculated by adding 2 points for each mismatch and 1 for each gap/ambiguity/clipped read										
Only the highest-scoring (i.e. 'worst') 2,000 positions are displayed in the table										
Pos.	Ref Base	Cons Base	Score	A	C	G	T	Gaps	Ambig	Clip'd
Mean coverage = 1.6, Standard Deviation = 0.8										
The following regions have NO coverage:										
From	To									
3401	3470									

Supplementary Figure 24: MacVector sequence report from AAV8$^{Ldlr\text{-}gA3}$ sanger sequence alignment. Positions of mismatches, if any, are displayed in the green highlight. Sanger reads are displayed as blue arrows.

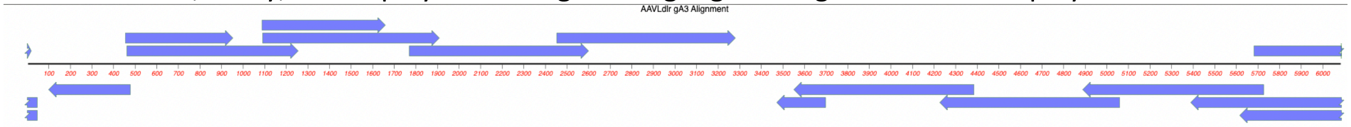

Potential sequencing problems report for AAVLdlr gA3 Alignment										
This tab is primarily designed to help you identify sequencing errors with genomic sequences and NGS data.										
Once you have a final sequence, run Align To Reference with it using a representative sample of your data.										
We recommend no more than 5 million reads unless you have more than 16 GB RAM. When you align, make sure										
you set the Hash Value to 12 and Sensitivity to 3 or less for optimal performance.										
Ref Base is the residue in the reference sequence										
Cons Base is the residue in the consensus sequence										
A, G, C and T are the counts of the occurence of each residue in reads that cross the location										
Gaps are the number of reads with gaps at that location										
Ambig are the number of reads with ambiguities at that location										
Clip'd are the number of reads that are clipped (greyed out) at that location										
Score is calculated by adding 2 points for each mismatch and 1 for each gap/ambiguity/clipped read										
Only the highest-scoring (i.e. 'worst') 2,000 positions are displayed in the table										
Pos.	Ref Base	Cons Base	Score	A	C	G	T	Gaps	Ambig	Clip'd
Mean coverage = 1.7, Standard Deviation = 0.8										
The following regions have NO coverage:										
From	To									
3279	3471									
The following regions have coverage less than two standard deviations from the mean (1):										
From	To									
3279	3471									

Supplementary Figure 25: MacVector sequence report from AAV8$^{Pcsk9\text{-}gA1}$ sanger sequence alignment. Positions of mismatches, if any, are displayed in the green highlight. Sanger reads are displayed as blue arrows.

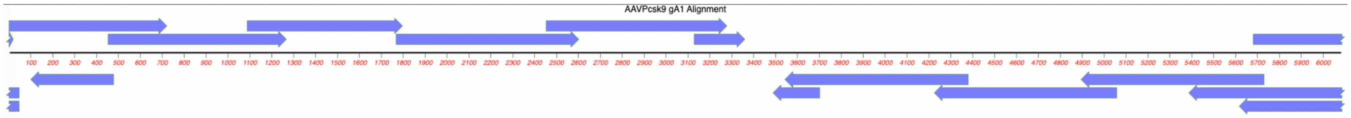

Potential sequencing problems report for AAVPcsk9 gA1 Alignment

This tab is primarily designed to help you identify sequencing errors with genomic sequences and NGS data.
Once you have a final sequence, run Align To Reference with it using a representative sample of your data.
We recommend no more than 5 million reads unless you have more than 16 GB RAM. When you align, make sure
you set the Hash Value to 12 and Sensitivity to 3 or less for optimal performance.

Ref Base is the residue in the reference sequence
Cons Base is the residue in the consensus sequence
A, G, C and T are the counts of the occurence of each residue in reads that cross the location
Gaps are the number of reads with gaps at that location
Ambig are the number of reads with ambiguities at that location
Clip'd are the number of reads that are clipped (greyed out) at that location
Score is calculated by adding 2 points for each mismatch and 1 for each gap/ambiguity/clipped read
Only the highest-scoring (i.e. 'worst') 2,000 positions are displayed in the table

Pos.	Ref Base	Cons Base	Score	A	C	G	T	Gaps	Ambig	Clip'd

Mean coverage = 1.5, Standard Deviation = 0.7

The following regions have NO coverage:

From	To
3359	3488

Supplementary Figure 26: MacVector sequence report from $AAV8^{Pcsk9\text{-}gA2}$ sanger sequence alignment. Positions of mismatches, if any, are displayed in the green highlight. Sanger reads are displayed as blue arrows.

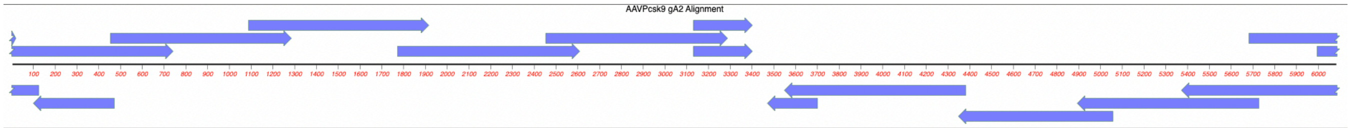

Potential sequencing problems report for AAVPcsk9 gA2 Alignment										
This tab is primarily designed to help you identify sequencing errors with genomic sequences and NGS data.										
Once you have a final sequence, run Align To Reference with it using a representative sample of your data.										
We recommend no more than 5 million reads unless you have more than 16 GB RAM. When you align, make sure										
you set the Hash Value to 12 and Sensitivity to 3 or less for optimal performance.										
Ref Base is the residue in the reference sequence										
Cons Base is the residue in the consensus sequence										
A, G, C and T are the counts of the occurence of each residue in reads that cross the location										
Gaps are the number of reads with gaps at that location										
Ambig are the number of reads with ambiguities at that location										
Clip'd are the number of reads that are clipped (greyed out) at that location										
Score is calculated by adding 2 points for each mismatch and 1 for each gap/ambiguity/clipped read										
Only the highest-scoring (i.e. 'worst') 2,000 positions are displayed in the table										
Pos.	Ref Base	Cons Base	Score	A	C	G	T	Gaps	Ambig	Clip'd
Mean coverage = 1.5, Standard Deviation = 0.6										
The following regions have NO coverage:										
From	To									
3401	3470									

Supplementary Figure 27: MacVector sequence report from AAV8$^{Pcsk9\text{-}gA3}$ sanger sequence alignment. Positions of mismatches, if any, are displayed in the green highlight. Sanger reads are displayed as blue arrows.

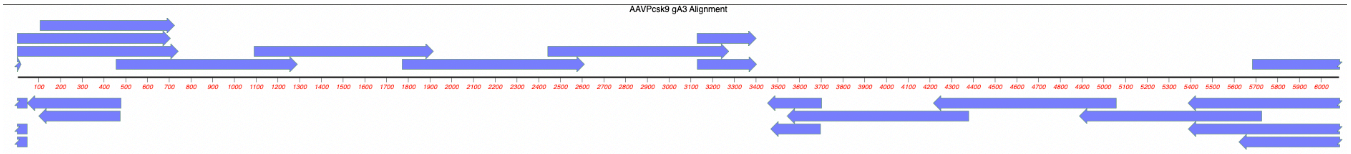

Potential sequencing problems report for AAVPcsk9 gA3 Alignment										
This tab is primarily designed to help you identify sequencing errors with genomic sequences and NGS data.										
Once you have a final sequence, run Align To Reference with it using a representative sample of your data.										
We recommend no more than 5 million reads unless you have more than 16 GB RAM. When you align, make sure										
you set the Hash Value to 12 and Sensitivity to 3 or less for optimal performance.										
Ref Base is the residue in the reference sequence										
Cons Base is the residue in the consensus sequence										
A, G, C and T are the counts of the occurence of each residue in reads that cross the location										
Gaps are the number of reads with gaps at that location										
Ambig are the number of reads with ambiguities at that location										
Clip'd are the number of reads that are clipped (greyed out) at that location										
Score is calculated by adding 2 points for each mismatch and 1 for each gap/ambiguity/clipped read										
Only the highest-scoring (i.e. 'worst') 2,000 positions are displayed in the table										
Pos.	Ref Base	Cons Base	Score	A	C	G	T	Gaps	Ambig	Clip'd
Mean coverage = 2.0, Standard Deviation = 1.3										
The following regions have NO coverage:										
From	To									
3402	3452									

Supplementary Table 1: Figure 1 significance tests. All significant *p*-values are bold and highlighted seafoam.

Figure	Target	Sex	Genotype	Approach	Statistic Method	P-Value
1e	*Ttr*	NA - mESC	$R26^{SAM/+}$	R26-targeted guide array	Pearson's Pearson's correlation coefficient	**0.994**
1f	*Rs1*	NA - mESC	$R26^{SAM/+}$	R26-targeted guide array	Pearson's Pearson's correlation coefficient	**0.996**
1g	*Alb*	NA - mESC	$R26^{SAM/+}$	R26-targeted guide array	Pearson's Pearson's correlation coefficient	**0.992**
1h	*Tsx*	NA - mESC	$R26^{SAM/+}$	R26-targeted guide array	Pearson's Pearson's correlation coefficient	**0.996**

Supplementary Table 2: Figure 2 significance tests. All significant *p*-values are bold and highlighted seafoam.

Figure	Target	Sex	Genotype	Approach	Statistic Method	P-Value	
2a	*Ttr*	Male	$R26^{SAM/+}$	R26-targeted guide array	One-tail, Student's unpaired *t*-test	Month 3	**0.000001**
						Month 4	**0.000001**
						Month 5	**0.000135**
						Month 6	**0.000001**
						Month 7	**0.000014**
2b	*Ttr*	Male	$R26^{SAM/+}$	R26-targeted guide array	One-tail, Student's unpaired *t*-test	Spleen	**0.000007**
						Lung	**0.000018**
						Pancreas	**0.000092**
						Kidney	**0.000133**
						Gonad	**0.000324**
						Muscle	**0.000867**
						Liver	**0.001235**
						Heart	**0.00311**
						Thymus	**0.003844**
						Brain	**0.026423**
2c	*Rs1*	Male	$R26^{SAM/+}$	R26-targeted guide array	One-tail, Student's unpaired *t*-test	Liver	**0.000001**
						Lung	**0.000002**
						Kidney	**0.000119**
						Spleen	**0.000535**
						Eye	**0.000792**
						Heart	**0.022224**
2d	*Ttr*	Male	$R26^{SAM/+}$	R26-targeted guide array	Pearson's correlation coefficient	Kidney	**0.99**
						Spleen	**0.99**
						Brain	**0.999**
2e	*Ttr/LacZ*	Female	$R26^{SAM/+}$	R26-targeted guide array	NA	NA	
2f	*Ttr/LacZ*	Female	$R26^{SAM/+}$	R26-targeted guide array	NA	NA	

Supplementary Table 3: Figure 3 significance tests. All significant *p*-values are bold and highlighted seafoam.

Figure	Target	Sex	Genotype	Approach	Titer/Dose	Statistic Method	P-Value	
3a	*Ttr*	Male	$R26^{SAM/+}$	3 guide array	2E+11	One-tail, Student's unpaired *t*-test	5 Days	**0.000424**
							19 Days	**0.000384**
							2 Months	**0.016572**
							3 Months	**0.029166**
							4 Months	**0.024172**
							5 Months	**0.000664**
							6 Months	**0.016092**
							7 Months	**0.013648**
							8 Months	0.114382
3b	*Tsx*	Male	$R26^{SAM/+}$	4 guide array	1E+11	One-tail, Student's unpaired *t*-test	Liver	**0.001026**
							Spleen	**0.01138**
							Kidney	0.27185
							Heart	0.115651
							Lung	0.21836

Figure	Target	Sex	Genotype	Approach	Titer/Dose	Statistic Method	P-Value	
3c	*Ttr*	Female	$R26^{SAM/SAM}$	3 guide array; individual guides	1E+11	One-tail, Student's unpaired *t*-test	SAM vs. GFP	0.3511
							SAM vs. 3 guide array	**0.0001**
							SAM vs. Guide gA1	**0.0001**
							SAM vs. Guide gA2	**0.0005**
							SAM vs. Guide gA3	**0.0001**
							3 guide array vs. SAM	**0.0001**
							3 guide array vs. GFP	**0.0001**
							3 guide array vs. Guide gA1	**0.0001**
							3 guide array vs. Guide gA2	**0.0001**
							3 guide array vs. Guide gA3	**0.0001**
3d	*Tsx*	Male	$R26^{SAM/SAM}$	4 guide array; individual guides	1.00E+11	One-tail, Student's unpaired *t*-test	Untreated vs. SAM Tsx Guide gA1	**0.0017**
							Untreated vs. SAM Tsx Guide gA2	**0.0125**
							Untreated vs. SAM Tsx Guide gA3	**0.0177**
							Untreated vs. SAM Tsx Guide gA4	**0.0004**
							Untreated vs. SAM Tsx 4 guide array	**0.0467**
							SAM Tsx 4 guide array vs. SAM Tsx Guide gA1	0.1081
							SAM Tsx 4 guide array vs. SAM Tsx Guide gA2	0.1374
							SAM Tsx 4 guide array vs. SAM Tsx Guide gA3	0.0562
							SAM Tsx4 guide array vs. SAM Tsx Guide gA4	**0.0471**
3e	*Ttr*	*Male*	$R26^{SAM/SAM}$	LNP Ttr-gA3	.5mpk	One-tail, Student's unpaired *t*-test	Day 1	**0.008075**
							Day 3	**0.002063**
							Day 7	**0.004157**
							Day 14	0.052138
							Day 21	**0.010346**
							Day 28	**0.041061**
							Day 35	**0.022289**
							Day 42	**0.00511**
							Day 49	**0.000023**
				LNP Ttr-gA3	1mpk	One-tail, Student's unpaired *t*-test	Day 1	**0.000018**
							Day 3	**0.000283**
							Day 7	**0.00216**
							Day 14	**0.001095**
							Day 21	**0.005414**
							Day 28	**0.014729**
							Day 35	**0.000247**
							Day 42	**0.000294**
							Day 49	**0.001486**
				LNP Ttr-gA3	2mpk	One-tail, Student's unpaired *t*-test	Day 1	**0.00307**
							Day 3	**0.000081**
							Day 7	**0.001023**
							Day 14	**0.000202**
							Day 21	**0.000292**
							Day 28	**0.000057**
							Day 35	**0.000595**
							Day 42	**0.007444**
							Day 49	**0.002646**
3f	*Ttr*	Male	$R26^{SAM/SAM}$	LNP Ttr-gA2	.5mpk	One-tail, Student's unpaired *t*-test	Day 0 vs Day 7	**0.000704**
							Day 0 vs Day 14	**0.000005**
							Day 0 vs Day 21	**0.000369**
							Day 0 vs Day 28	**0.022395**
							Day 0 vs Day 35	0.449093
							Day 0 vs Day 42	0.305166
							Day 0 vs Day 49	0.167872
							Day 0 vs Day 56	0.251797
							Day 0 vs Day 63	0.073351
				LNP Ttr-gA2	.5mpk	One-tail, Student's unpaired *t*-test	Day 28 vs Day 7	0.477549
							Day 28 vs Day 14	0.60848
							Day 28 vs Day 21	0.594508
							Day 28 vs Day 28	0.885534
							Day 28 vs Day 35	**0.000036**
							Day 28 vs Day 42	**0.000093**
							Day 28 vs Day 49	**0.005597**
							Day 28 vs Day 56	0.223234
							Day 28 vs Day 63	**0.037638**
				LNP Ttr-gA2	.5mpk	One-tail, Student's unpaired *t*-test	Day 0 and 28 vs Day 7	**0.000766**
							Day 0 and 28 vs Day 14	**0.000026**
							Day 0 and 28 vs Day 21	**0.00506**
							Day 0 and 28 vs Day 28	**0.031024**
							Day 0 and 28 vs Day 35	**0.011233**
							Day 0 and 28 vs Day 42	**0.000156**
							Day 0 and 28 vs Day 49	**0.000102**
							Day 0 and 28 vs Day 56	**0.006938**
							Day 0 and 28 vs Day 63	0.052062

Figure	Target	Sex	Genotype	Approach	Titer/Dose	Statistic Method	P-Value	
3g	*Ttr*	Male	R26$^{SAM/SAM}$	3 guide AAV array; 3 guide array plasmid, Ubi Ttr cDNA	2e11 virus; 50ug plasmid	One-tail, Student's unpaired *t*-test	Untreated vs Array Baseline	0.757315
							Untreated vs Array week 1	**0.000001**
							Untreated vs Array week 3	**0.000001**
							Untreated vs HDD plasmid Baseline	0.224205
							Untreated vs HDD plasmid week 1	**0.002222**
							Untreated vs HDD plasmid week 3	0.110687
							Untreated vs HDD plasmid gRNA Baseline	0.284077
							Untreated vs HDD plasmid gRNA week 1	**0.003585**
							Untreated vs HDD plasmid gRNA week 3	0.6615
3h	*Ngn3/Btc*	Female	R26$^{SAM/SAM}$	4 guide array	2E+11	One-tail, Student's unpaired *t*-test	Liver (Ngn3)	**0.001234**
							Spleen (Ngn3)	0.428402
							Liver (Btc)	**0.001969**
							Spleen (Btc)	0.26044

Supplementary Table 4: Figure 4 significance tests. All significant *p*-values are bold and highlighted seafoam.

Figure	Target	Sex	Genotype	Approach		Titer	Statistic Method	P-Value	
4a	*Ttr*	Male	R26$^{SAM/+}$	3 guide array	LNP Cre	2E+11	One-tail, Student's unpaired *t*-test	WT vs LSL SAM LNP CRE AAV8 3 guide array	**0.0001**
								WT vs LSL SAM LNP Cre	0.9996
								WT vs LSL SAM AAV8 3 guide array	0.9994
								WT vs LSL SAM	0.9998
								WT vs SAM LNP Cre	0.9996
								WT vs SAM AAV8 3 guide array	**0.0001**
								WT vs SAM	0.9996
							One-tail, Student's unpaired *t*-test	LSL SAM vs LSL SAM LNP CRE AAV8 3 guide array	**0.0001**
								LSL SAM vs LSL SAM LNP Cre	0.9975
								LSL SAM vs LSL SAM AAV8 3 guide array	0.9997
								LSL SAM vs WT	0.9994
								LSL SAM vs SAM LNP Cre	**0.0001**
								LSL SAM vs SAM AAV8 3 guide array	0.9977
								LSL SAM vs SAM	0.9998
4b	*Ttr*	Male	*R26*$^{SAM/+}$	3 guide array	LNP Cre	2.00E+11	NA		
4c	Tsx	Male	R26$^{SAM/+}$	R26-targeted guide array	LNP Cre	NA	One-tail, Student's unpaired *t*-test	LSL SAM PBS vs LSL SAM LNP Cre Liver	**0.000082**
								LSL SAM PBS vs LSL SAM LNP Cre Kidney	0.159769
								LSL SAM PBS vs LSL SAM LNP Cre testes	0.17354
								LSL SAM PBS vs LSL SAM LNP Cre Heart	0.176737
								LSL SAM PBS vs LSL SAM LNP Cre Spleen	0.145058
								LSL SAM PBS vs LSL SAM LNP Cre Lung	0.206547
4d	*Ttr*	Male	*R26*$^{SAM/SAM}$	3 guide array		1.20E+09	NA		
4e	*Ttr*	Male	*R26*$^{SAM/SAM}$	3 guide array		1.20E+09	NA		
4f	*Ldlr/Pcsk9 /Ttr*	Neonate	*R26*$^{SAM/SAM}$	3 guide array		1.00E+11	NA		
4g	*Ldlr/Pcsk9 /Ttr*	Neonate	*R26*$^{SAM/SAM}$	3 guide array		1E+11	One-tail, Student's unpaired *t*-test	TTR	**0.0015**
								LDLR	**0.001395**
								PCSK9	**0.000052**

Supplementary Table 5: Figure 5 significance tests. All significant *p*-values are bold and highlighted seafoam.

Figure	Target	Sex	Genotype	Approach	Titer	Statistic Method	P-Value	
5a	*Pcsk9:* LDL	Male	R26$^{SAM/SAM}$	3 guide array; individual guides	1E+11	One-tail, Student's unpaired t-test	3 guide array 2 wks	**0.000039**
							3 guide array 4 wks	**0.000817**
							3 guide array 6 wks	**0.01079**
							3 guide array 8 wks	**0.037423**
							3 guide array 12 wks	0.109931
							Guide gA1 2 wks	**0.005337**
							Guide gA1 4 wks	**0.000284**
							Guide gA1 6 wks	**0.001164**
							Guide gA1 8 wks	**0.002705**
							Guide gA1 12 wks	**0.001007**
							Guide gA2 2 wks	**0.007157**
							Guide gA2 4 wks	0.075126
							Guide gA2 6 wks	0.206202
							Guide gA2 8 wks	**0.001732**
							Guide gA2 12 wks	0.474722
							Guide gA3 2 wks	0.051889
							Guide gA3 4 wks	**0.001848**
							Guide gA3 6 wks	**0.004423**
							Guide gA3 8 wks	0.060279
							Guide gA3 12 wks	**0.038732**
5b	*Pcsk9:* Total Cholesterol	Male	R26$^{SAM/SAM}$	3 guide array; individual guides	1E+11	One-tail, Student's unpaired t-test	3 guide array 2 wks	**0.000038**
							3 guide array 4 wks	**0.001453**
							3 guide array 6 wks	0.163627
							3 guide array 8 wks	0.378898
							3 guide array 12 wks	0.118781
							Guide gA1 2 wks	**0.001428**
							Guide gA1 4 wks	**0.000164**
							Guide gA1 6 wks	**0.003062**
							Guide gA1 8 wks	**0.019129**
							Guide gA1 12 wks	**0.004269**
							Guide gA2 2 wks	**0.012377**
							Guide gA2 4 wks	**0.008214**
							Guide gA2 6 wks	0.245205
							Guide gA2 8 wks	0.667286
							Guide gA2 12 wks	0.052172
							Guide gA3 2 wks	**0.000696**
							Guide gA3 4 wks	**0.000098**
							Guide gA3 6 wks	**0.004916**
							Guide gA3 8 wks	0.18693
							Guide gA3 12 wks	**0.002322**
5c	*Pcsk9:* HDL	Male	R26$^{SAM/SAM}$	3 guide array; individual guides	1E+11	One-tail, Student's unpaired t-test	3 guide array 2 wks	**0.046908**
							3 guide array 4 wks	**0.027043**
							3 guide array 6 wks	**0.016825**
							3 guide array 8 wks	**0.005334**
							3 guide array 12 wks	**0.007955**
							Guide gA1 2 wks	**0.024768**
							Guide gA1 4 wks	0.081164
							Guide gA1 6 wks	0.173709
							Guide gA1 8 wks	0.13866
							Guide gA1 12 wks	**0.029702**
							Guide gA2 2 wks	**0.002147**
							Guide gA2 4 wks	**0.004767**
							Guide gA2 6 wks	**0.014386**
							Guide gA2 8 wks	**0.005793**
							Guide gA2 12 wks	**0.019755**
							Guide gA3 2 wks	**0.000753**
							Guide gA3 4 wks	**0.000279**
							Guide gA3 6 wks	**0.000413**
							Guide gA3 8 wks	**0.012972**
							Guide gA3 12 wks	0.191463

Figure	Target	Sex	Genotype	Approach	Titer	Statistic Method	P-Value	
5d	*Pcsk9:* LDL	Female	Female	3 guide array; individual guides	1E+11	One-tail, Student's unpaired t-test	3 guide array 2 wks	**0.004472**
							3 guide array 4 wks	**0.0002**
							3 guide array 6 wks	**0.000003**
							Guide gA1 2 wks	**0.016494**
							Guide gA1 4 wks	**0.002686**
							Guide gA1 6 wks	**0.000081**
							Guide gA2 2 wks	0.411517
							Guide gA2 4 wks	0.523041
							Guide gA2 6 wks	0.445811
							Guide gA3 2 wks	0.063334
							Guide gA3 4 wks	0.063044
							Guide gA3 6 wks	0.110637
5e	*Pcsk9:* Total Cholesterol	Female	$R26^{SAM/SAM}$	3 guide array; individual guides	1E+11	One-tail, Student's unpaired t-test	3 guide array 2 wks	**0.000041**
							3 guide array 4 wks	**0.006862**
							3 guide array 6 wks	**0.003203**
							Guide gA1 2 wks	**0.007246**
							Guide gA1 4 wks	0.071149
							Guide gA1 6 wks	**0.003658**
							Guide gA2 2 wks	0.443229
							Guide gA2 4 wks	0.640932
							Guide gA2 6 wks	0.530164
							Guide gA3 2 wks	0.215271
							Guide gA3 4 wks	0.106639
							Guide gA3 6 wks	0.096226
5f	*Pcsk9:* HDL	Female	$R26^{SAM/SAM}$	3 guide array; individual guides	1E+11	One-tail, Student's unpaired t-test	3 guide array 2 wks	**0.000686**
							3 guide array 4 wks	0.072985
							3 guide array 6 wks	0.462284
							Guide gA1 2 wks	**0.00932**
							Guide gA1 4 wks	0.506012
							Guide gA1 6 wks	0.289599
							Guide gA2 2 wks	0.385162
							Guide gA2 4 wks	0.638359
							Guide gA2 6 wks	0.246705
							Guide gA3 2 wks	0.378268
							Guide gA3 4 wks	0.071083
							Guide gA3 6 wks	0.219194
5g	*Ldlr:* LDL	Female	$R26^{SAM/SAM}$	3 guide array; individual guides; 2 guide array	1.00E+11	One-tail, Student's unpaired t-test	3 guide array 9 wks	**0.000535**
							3 guide array 11 wks	0.066307
							3 guide array 13 wks	0.694182
							3 guide array 15 wks	0.961148
							Guide gA1 9 wks	**0.000135**
							Guide gA1 11 wks	0.206993
							Guide gA1 13 wks	0.329522
							Guide gA1 15 wks	0.901845
							Guide gA2 9 wks	0.349503
							Guide gA2 11 wks	**0.003095**
							Guide gA2 13 wks	0.058393
							Guide gA2 15 wks	0.08652
							Guide gA3 9 wks	**0.039939**
							Guide gA3 11 wks	0.183413
							Guide gA3 13 wks	0.209094
							Guide gA3 15 wks	0.627369
							2 guide array 9 wks	**0.000024**
							2 guide array 11 wks	**0.001387**
							2 guide array 13 wks	**0.000351**
							2 guide array 15 wks	**0.020585**

Figure	Target	Sex	Genotype	Approach	Titer	Statistic Method	P-Value	
5h	*Ldlr:* Total Cholesterol	Female	$R26^{SAM/SAM}$	3 guide array; individual guides; 2 guide array	1.00E+11	One-tail, Student's unpaired t-test	3 guide array 9 wks	**0.001766**
							3 guide array 11 wks	0.203639
							3 guide array 13 wks	0.841979
							3 guide array 15 wks	0.897055
							Guide gA1 9 wks	0.072805
							Guide gA1 11 wks	0.215229
							Guide gA1 13 wks	0.693373
							Guide gA1 15 wks	0.915434
							Guide gA2 9 wks	0.47272
							Guide gA2 11 wks	**0.000279**
							Guide gA2 13 wks	**0.00372**
							Guide gA2 15 wks	**0.035747**
							Guide gA3 9 wks	0.217531
							Guide gA3 11 wks	0.297848
							Guide gA3 13 wks	0.724858
							Guide gA3 15 wks	0.53275
							2 guide array 9 wks	**0.001126**
							2 guide array 11 wks	**0.000483**
							2 guide array 13 wks	**0.001585**
							2 guide array 15 wks	**0.001154**
5i	*Ldlr*: HDL	Female	$R26^{SAM/SAM}$	3 guide array; individual guides; 2 guide array	1.00E+11	One-tail, Student's unpaired t-test	3 guide array 9 wks	**0.01128**
							3 guide array 11 wks	0.097005
							3 guide array 13 wks	0.978477
							3 guide array 15 wks	0.847388
							Guide gA1 9 wks	0.150334
							Guide gA1 11 wks	0.143599
							Guide gA1 13 wks	0.530653
							Guide gA1 15 wks	0.63125
							Guide gA2 9 wks	0.191707
							Guide gA2 11 wks	**0.000044**
							Guide gA2 13 wks	**0.003364**
							Guide gA2 15 wks	**0.027216**
							Guide gA3 9 wks	0.868116
							Guide gA3 11 wks	**0.028208**
							Guide gA3 13 wks	0.507891
							Guide gA3 15 wks	0.051571
							2 guide array 9 wks	**0.004049**
							2 guide array 11 wks	**0.000652**
							2 guide array 13 wks	**0.00473**
							2 guide array 15 wks	**0.001832**
5j	*Ldlr:* LDL	Male	$R26^{SAM/SAM}$	3 guide array; individual guides; 2 guide array	1E+11	One-tail, Student's unpaired t-test	Guide gA1 8 wks	**0.002945**
							Guide gA1 10 wks	**0.001644**
							Guide gA2 8 wks	0.393089
							Guide gA2 10 wks	0.228807
							Guide gA3 8 wks	0.632307
							Guide gA3 10 wks	0.824147
							2 guide array 8 wks	**0.000055**
							2 guide array 10 wks	**0.00001**
5k	*Ldlr:* Total Cholesterol	Male	$R26^{SAM/SAM}$	3 guide array; individual guides; 2 guide array	1E+11	One-tail, Student's unpaired t-test	Guide gA1 8 wks	**0.00067**
							Guide gA1 10 wks	**0.003406**
							Guide gA2 8 wks	0.184293
							Guide gA2 10 wks	0.147489
							Guide gA3 8 wks	0.29458
							Guide gA3 10 wks	0.432315
							2 guide array 8 wks	**0.000001**
							2 guide array 10 wks	**0.000001**
5l	*Ldlr*	Male	$R26^{SAM/SAM}$	3 guide array; individual guides; 2 guide array	1E+11	One-tail, Student's unpaired t-test	Guide gA1 8 wks	**0.002995**
							Guide gA1 10 wks	**0.012994**
							Guide gA2 8 wks	0.147528
							Guide gA2 10 wks	0.273975
							Guide gA3 8 wks	0.248758
							Guide gA3 10 wks	0.313028
							2 guide array 8 wks	**0.000081**
							2 guide array 10 wks	**0.000004**

Supplementary Table 6: Figure 6 significance tests. All significant *p*-values are bold and highlighted seafoam.

Figure	Target	Sex	Genotype	Approach	Titer	Statistic Method	P-Value	
6a	*hTTR*	NA	NA	3 guide array	NA	NA		
6b	*hTTR*	NA	NA	NA	NA	One-tail, Student's unpaired t-test	Humanized vs NHS	**0.0071**
6c	*hTTR*	Mixed	$R26^{SAM/SAM}$	3 guide array	2.00E+11	One-tail, Student's unpaired t-test	baseline vs treated	**0.0365**

Supplementary Table 7: Supplementary Figure S1 significance tests. All significant *p*-values are bold and highlighted seafoam.

Figure	Target	Sex	Genotype	Approach	Statistic Method	P-Value	
S1a	*Tsx*	NA - mESC	$R26^{SAM/+}$	R26-targeted guide array	One-tail, Student's unpaired t-test	*Chic1*	0.06885
						Tsx	**0.00031**
						Xist	0.36724
S1b	*Ttr*	NA - mESC	$R26^{SAM/+}$	R26-targeted guide array	One-tail, Student's unpaired t-test	*Ttr v SAM*	**5E-06**
	Celrr					*Celrr v SAM*	**1.4E-05**
	Rs1					*Rs1 v SAM*	**0.0481**
	Alb					*Alb v SAM*	**0.00136**
	Pd-l1					*Pd-l1 v SAM*	**1E-06**
	Tmem97					*Tmem97 v SAM*	**1E-06**
S1c	SAM	NA - mESC	$R26^{SAM/+}$	R26-targeted guide array	Pearson's Pearson's correlation coefficient	*SAM vs LSL-SAM*	**0.994**
	LSL-SAM					*LSL-SAM vs WT*	**0.998**
S1d	*Mixed*	NA - mESC	$R26^{SAM/+}$	R26-targeted guide array	NA	*NA*	NA
S1e	*Rs1*	NA - mESC	$R26^{SAM/+}$	R26-targeted guide array	One-tail, Student's unpaired t-test	*Ppef1*	**0.00154**
						Rs1	**1E-06**
						Cdkl5	0.4255
S1f	*Alb*	NA - mESC	$R26^{SAM/+}$	R26-targeted guide array	One-tail, Student's unpaired t-test	*Ankrd17*	0.08432
						Alb	**1E-06**
						Afp	0.06435
S1g	*Ttr*	NA - mESC	$R26^{SAM/+}$	R26-targeted guide array	One-tail, Student's unpaired t-test	*Dsg2*	0.20024
						Ttr	**0.00014**
						B4Galt6	0.1873

Supplementary Table 8: Supplementary Figure S2 significance tests. All significant *p*-values are bold and highlighted seafoam.

Figure	Target	Sex	Genotype	Approach	Statistic Method	P-Value	
s2a	dCas9 SAM	Male	*R26$^{SAM/+}$*	R26-targeted guide array	NA		
s2b	dCas9 SAM	Male	*R26$^{SAM/+}$*	R26-targeted guide array	NA		
s2c	*Ttr*	Male	*R26$^{SAM/+}$*	R26-targeted guide array	NA		
s2d	*Ttr*	Male	R26$^{SAM/+}$	R26-targeted guide array	One-tail, Student's unpaired t-test	Muscle	**0.000018**
						Spleen	**0.000007**
						Kidney	**0.01061**
						Brain	**0.001579**
						Liver	**0.000001**
s2e	*Rs1*	Male	*R26$^{SAM/+}$*	R26-targeted guide array	NA		
s2f	*Ppef1*	Male	R26$^{SAM/+}$	R26-targeted guide array	One-tail, Student's unpaired t-test	Liver	**0.000084**
						Kidney	**0.000163**
						Lung	**0.000405**
						spleen	**0.000032**
						Heart	**0.000158**
						Eye	**0.000004**
s2g	*Cdkl5*	Male	R26$^{SAM/+}$	R26-targeted guide array	One-tail, Student's unpaired t-test	Liver	**0.000524**
						Kidney	**0.000022**
						Lung	**0.001895**
						spleen	**0.000024**
						Heart	**0.003981**
						Eye	**0.000067**
s2h	*Ppef1*	Male	*R26$^{SAM/+}$*	R26-targeted guide array	NA		
s2i	*Cdkl5*	Male	*R26$^{SAM/+}$*	R26-targeted guide array	NA		

Supplementary Table 9: Supplementary Figure S3 significance tests. All significant *p*-values are bold and highlighted seafoam.

Figure	Target	Sex	Genotype	Approach	Statistic Method	P-Value	
s3a	*Ttr*	Male	R26$^{SAM/+}$ vs *R26$^{+/+}$*	NA	Pearson's Pearson's correlation coefficient	Liver	**0.999**
						Spleen	**0.993**
						Brain	**0.999**
						Muscle	**0.997**
						Kidney	**0.999**
s3b	*Ttr*	Male	R26$^{SAM/+}$	R26-targeted guide array	Pearson's Pearson's correlation coefficient	Liver	**0.991**
						Muscle	**0.993**
s3c	*Ttr*	Male	R26$^{SAM/+}$	R26-targeted guide array	One-tail, Student's unpaired t-test	Dsg	0.200237
						Ttr	**0.00014**
						B4Galt6	0.187297

Supplementary Table 10: Supplementary Figure S4 significance tests. All significant *p*-values are bold and highlighted seafoam.

Figure	Target	Sex	Genotype	Approach	Titer	Statistic Method
s4a	NA	NA	NA	NA	NA	NA
s4b	NA	NA	NA	NA	NA	NA
s4c	NA	NA	NA	NA	NA	NA
s4d	Ttr	Male	*R26* $^{SAM/+}$	3 guide array	2E+11	NA
s4e	NA	NA				NA
s4f	NA	NA				NA

Supplementary Table 11: Supplementary Figure S5 significance tests. All significant *p*-values are bold and highlighted seafoam.

Figure	Target	Sex	Genotype	Approach	Titer	Statistic Method	P-Value	
s5a	*Ttr*	Male	*R26* $^{SAM/SAM}$	3 guide array	1.20E+09	NA	NA	
s5b	*Ttr*	Male	*R26* $^{SAM/SAM}$	3 guide array	1.00E+11	One-tail, Student's unpaired t-test	0.8599	
s5c	*Ttr*	Female	*R26* $^{SAM/SAM}$	3 guide array	1.00E+11	One-tail, Student's unpaired t-test	0.1892	
s5d	*Ttr*	Male	R26$^{SAM/SAM}$	LNP Ttr-gA2	.5mpk	One-tail, Student's unpaired t-test	Day 0 vs Day 7	**0.000704**
							Day 0 vs Day 14	**0.000005**
							Day 0 vs Day 21	**0.000369**
							Day 0 vs Day 28	**0.022395**
							Day 0 vs Day 35	0.449093
							Day 0 vs Day 42	0.305166
							Day 0 vs Day 49	0.167872
							Day 0 vs Day 56	0.251797
							Day 0 vs Day 63	0.073351
							Day 14 vs Day 7	0.242878
							Day 14 vs Day 14	0.998971
							Day 14 vs Day 21	**0.004461**
							Day 14 vs Day 28	**0.000409**
							Day 14 vs Day 35	**0.00014**
							Day 14 vs Day 42	**0.001218**
							Day 14 vs Day 49	**0.029656**
							Day 14 vs Day 56	0.394798
							Day 14 vs Day 63	0.537053
							Day 0 and 14 vs Day 7	**0.000015**
							Day 0 and 14 vs Day 14	**0.000199**
							Day 0 and 14 vs Day 21	**0.000422**
							Day 0 and 14 vs Day 28	**0.000151**
							Day 0 and 14 vs Day 35	**0.006947**
							Day 0 and 14 vs Day 42	**0.025224**
							Day 0 and 14 vs Day 49	**0.022041**
							Day 0 and 14 vs Day 56	0.585655
							Day 0 and 14 vs Day 63	0.249726
s5e	*Ttr*	Male	*R26* $^{SAM/SAM}$	3 guide array	1.20E+09	NA		
s5f	*Ttr*	Male	*R26* $^{SAM/SAM}$	3 guide array	1.20E+09	NA		

Supplementary Table 12: Supplementary Figure S6 significance tests. All significant *p*-values are bold and highlighted seafoam.

Figure	Target	Sex	Genotype	Approach	Titer	Statistic Method	P-Value	
s6a	*Pcsk9*	NA				NA		
s6b	*Pcsk9*: LDL	Male	*R26* $^{SAM/SAM}$	3 guide array; individual guides	1.00E+11	One-tail, Student's unpaired t-test	3 guide array, 2 weeks	**0.003667**
							3 guide array, 4 weeks	**0.002869**
							3 guide array, 6 weeks	**0.004111**
							3 guide array, 8 weeks	**0.006974**
							3 guide array, 12 weeks	**0.047547**
							Guide gA1, 2 weeks	**0.007644**
							Guide gA1, 4 weeks	**0.00397**
							Guide gA1, 6 weeks	**0.008657**
							Guide gA1, 8 weeks	**0.001225**
							Guide gA1, 12 weeks	**0.000552**
							Guide gA2, 2 weeks	**0.014163**
							Guide gA2, 4 weeks	0.276802
							Guide gA2, 6 weeks	0.098715
							Guide gA2, 8 weeks	**0.00515**
							Guide gA2, 12 weeks	0.069659
							Guide gA3, 2 weeks	**0.006389**
							Guide gA3, 4 weeks	**0.000066**
							Guide gA3, 6 weeks	**0.002295**
							Guide gA3, 8 weeks	0.186816
							Guide gA3, 12 weeks	**0.031195**
s6c	*Pcsk9*: Triglycerides	Male	*R26* $^{SAM/SAM}$	3 guide array; individual guides	1.00E+11	One-tail, Student's unpaired t-test	3 guide array 2 wks	0.875584
							3 guide array 4 wks	**0.016058**
							3 guide array 6 wks	**0.045184**
							3 guide array 8 wks	0.071454
							3 guide array 12 wks	0.050323
							Guide gA1 2 wks	0.307529
							Guide gA1 4 wks	0.930472
							Guide gA1 6 wks	0.471818
							Guide gA1 8 wks	0.943868
							Guide gA1 12 wks	0.140392
							Guide gA2 2 wks	0.215957
							Guide gA2 4 wks	**0.022565**
							Guide gA2 6 wks	**0.001941**
							Guide gA2 8 wks	**0.008691**
							Guide gA2 12 wks	**0.006743**
							Guide gA3 2 wks	0.367837
							Guide gA3 4 wks	0.926086
							Guide gA3 6 wks	0.568467
							Guide gA3 8 wks	0.747687
							Guide gA3 12 wks	0.574367
s6d	*Pcsk9*	Male	*R26* $^{SAM/SAM}$	3 guide array	1.00E+11	One-tail, Student's unpaired t-test	3 guide array	0.17785
							Guide gA1	**0.003803**
							Guide gA2	0.157617
							Guide gA3	**0.015013**
s6e	*Pcsk9*: Triglycerides	Female	*R26* $^{SAM/SAM}$	3 guide array; individual guides	1.00E+11	One-tail, Student's unpaired t-test	3 guide array 2 wks	0.116591
							3 guide array 4 wks	**0.010281**
							3 guide array 6 wks	0.595317
							Guide gA1 2 wks	0.083687
							Guide gA1 4 wks	**0.03786**
							Guide gA1 6 wks	**0.043623**
							Guide gA2 2 wks	0.283649
							Guide gA2 4 wks	**0.043125**
							Guide gA2 6 wks	0.289164
							Guide gA3 2 wks	0.221599
							Guide gA3 4 wks	0.247244
							Guide gA3 6 wks	**0.019767**

Figure	Target	Sex	Genotype	Approach	Titer	Statistic Method	P-Value	
s6f	serum *Pcsk9*	Female	$R26^{SAM/SAM}$	3 guide array; individual guides	1.00E+11	One-tail, Student's unpaired t-test	3 guide array 6 wks	**0.012593**
							3 guide array 10 wks	0.15711
							3 guide array 12 wks	0.055948
							Guide gA1 6 wks	**0.025716**
							Guide gA1 10 wks	**0.014055**
							Guide gA1 12 wks	**0.021179**
							Guide gA2 6 wks	0.919114
							Guide gA2 10 wks	0.997466
							Guide gA2 12 wks	0.777283
							Guide gA3 6 wks	0.116897
							Guide gA3 10 wks	**0.028167**
							Guide gA3 12 wks	0.175753

Supplementary Table 13: Supplementary Figure S7 significance tests. All significant *p*-values are bold and highlighted seafoam.

Figure	Target	Sex	Genotype	Approach	Titer	Statistic Method	P-Value	
s7a	*Ldlr*	NA				NA	NA	
s7b	*Ldlr*: LDL	Female	$R26^{SAM/SAM}$	3 guide array; individual guides	1.00E+11	One-tail, Student's unpaired t-test	3 guide array 8 wks	**0.026723**
							3 guide array 10 wks	0.682449
							3 guide array 12 wks	0.509784
							Guide gA1 8 wks	**0.002388**
							Guide gA1 10 wks	**0.004297**
							Guide gA1 12 wks	**0.00957**
							Guide gA2 8 wks	**0.011222**
							Guide gA2 10 wks	0.113586
							Guide gA2 12 wks	0.262667
							Guide gA3 8 wks	0.198351
							Guide gA3 10 wks	**0.028962**
							Guide gA3 12 wks	0.34244
s7c	*Ldlr*	Female	$R26^{SAM/SAM}$	3 guide array; individual guides	1E+11	One-tail, Student's unpaired t-test	3 guide array_0 to 8	**0.035702**
							3 guide array_6 to 8	**0.000001**
							Guide gA1_0 to 8	**0.000045**
							Guide gA1_6 to 8	**0.000265**
							Guide gA2_0 to 8	**0.002865**
							Guide gA2_6 to 8	**0.000004**
							Guide gA3_0 to 8	0.374072
							Guide gA3_6 to 8	**0.006082**
s7d	*Ldlr:* Total cholesterol	Female	$R26^{SAM/SAM}$	3 guide array; individual guides	1.00E+11	One-tail, Student's unpaired t-test	3 guide array 8 wks	**0.007107**
							3 guide array 10 wks	0.983822
							3 guide array 12 wks	0.901777
							Guide gA1 8 wks	**0.000253**
							Guide gA1 10 wks	**0.000503**
							Guide gA1 12 wks	**0.002132**
							Guide gA2 8 wks	**0.001526**
							Guide gA2 10 wks	**0.021809**
							Guide gA2 12 wks	**0.035373**
							Guide gA3 8 wks	0.384988
							Guide gA3 10 wks	0.080086
							Guide gA3 12 wks	0.4833
s7e	*Ldlr*: HDL	Female	$R26^{SAM/SAM}$	3 guide array; individual guides	1.00E+11	One-tail, Student's unpaired t-test	3 guide array 8 wks	**0.002243**
							3 guide array 10 wks	0.476451
							3 guide array 12 wks	0.769896
							Guide gA1 8 wks	**0.000027**
							Guide gA1 10 wks	**0.00018**
							Guide gA1 12 wks	0.190316
							Guide gA2 8 wks	**0.000127**
							Guide gA2 10 wks	0.064338
							Guide gA2 12 wks	0.320208
							Guide gA3 8 wks	**0.043658**
							Guide gA3 10 wks	**0.012809**
							Guide gA3 12 wks	0.136939

Figure	Target	Sex	Genotype	Approach	Titer	Statistic Method	P-Value	
s7f	*Ldlr:* Triglycerides	Female	*$R26^{SAM/SAM}$*	3 guide array; individual guides	1.00E+11	One-tail, Student's unpaired t-test	3 guide array 8 wks	0.570692
							3 guide array 10 wks	0.649406
							3 guide array 12 wks	0.091516
							Guide gA1 8 wks	**0.012756**
							Guide gA1 10 wks	0.108444
							Guide gA1 12 wks	0.263486
							Guide gA2 8 wks	0.338268
							Guide gA2 10 wks	0.129215
							Guide gA2 12 wks	0.724361
							Guide gA3 8 wks	0.781191
							Guide gA3 10 wks	0.335807
							Guide gA3 12 wks	0.154306
s7g	*Ldlr*	Female	*$R26^{SAM/SAM}$*	3 guide array; individual guides	1.00E+11	One-tail, Student's unpaired t-test	3 guide array	0.080743
							Guide gA1	**0.000002**
							Guide gA2	0.193167
							Guide gA3	0.143169
s7h	*Ldlr*	Female	*$R26^{SAM/SAM}$*	2 guide array; 3 guide array; individual guides	1.00E+11	One-tail, Student's unpaired t-test	3 guide array_0 to 8	**0.000752**
							3 guide array_6 to 8	0.107846
							Guide gA1_0 to 8	**0.000088**
							Guide gA1_6 to 8	**0.013041**
							Guide gA2_0 to 8	0.147817
							Guide gA2_6 to 8	0.29162
							Guide gA3_0 to 8	0.461584
							Guide gA3_6 to 8	**0.042301**
							2 guide array_0 to 8	**0.019652**
							2 guide array_6 to 8	**0.008036**
s7i	*Ldlr*	Female	*$R26^{SAM/SAM}$*	2 guide array; 3 guide array; individual guides	1.00E+11	One-tail, Student's unpaired t-test	3 guide array	**0.0067**
							Guide gA1	0.0648
							Guide gA2	**0.0351**
							Guide gA3	0.7015
							2 guide array	**0.0029**
s7j	Ldlr: Triglycerides	Female	$R26^{SAM/SAM}$	2 guide array; 3 guide array; individual guides	1E+11	One-tail, Student's unpaired t-test	3 guide array 9 wks	0.204817
							3 guide array 11 wks	0.226103
							3 guide array 13 wks	0.272435
							3 guide array 15 wks	0.37748
							Guide gA1 9 wks	0.55654
							Guide gA1 11 wks	0.555939
							Guide gA1 13 wks	**0.038909**
							Guide gA1 15 wks	0.786844
							Guide gA2 9 wks	0.206255
							Guide gA2 11 wks	0.324626
							Guide gA2 13 wks	**0.018611**
							Guide gA2 15 wks	0.842203
							Guide gA3 9 wks	0.871984
							Guide gA3 11 wks	0.580322
							Guide gA3 13 wks	0.493948
							Guide gA3 15 wks	0.396028
							2 guide array 9 wks	**0.037636**
							2 guide array 11 wks	0.270443
							2 guide array 13 wks	0.41835
							2 guide array 15 wks	0.93378
s7k	*Ldlr:* Triglycerides	Female	*$R26^{SAM/SAM}$*	2 guide array; individual guides	1.00E+11	One-tail, Student's unpaired t-test	Guide gA1 8 wks	0.55654
							Guide gA1 10 wks	0.555939
							Guide gA2 8 wks	0.751451
							Guide gA2 10 wks	0.360126
							Guide gA3 8 wks	**0.005016**
							Guide gA3 10 wks	0.803918
							2 guide array 8 wks	0.493948
							2 guide array 10 wks	0.396028

Supplementary Table 14: dCas9 SAM target specific protospacer sequences

Guide	Sequence
**Ttr gA1**	ACGGUUGCCCUCUUUCCCAA
**Ttr gA2**	ACUGUCAGACUCAAAGGUGC
**Ttr gA3**	GACAAUAAGUAGUCUUACUC
**Pcsk9 gA1**	GAAGAGUCAUGGGUCACAGG
**Pcsk9 gA2**	CAGGCGGGGUGCCAACUCAG
**Pcsk9 gA3**	AAUAUUAACUAACUUCUCCU
**Ldlr gA1**	AAGCGGUGAAAUUCUGUGGG
**Ldlr gA2**	CACUCAAACAGCAACGCGGG
**Ldlr gA3**	CUUCACCUCACUGAGCGGGG
**Ngn3 gA1**	CACAGCUGGAUUCCGGACAA
**Ngn3 gA2**	ACUCCACGCCUCCCCCGUCG
**Btc gA1**	GUCAGGUGAGACCCAGCAGG
**Btc gA2**	GACACGCUCAGAGGGCCACG
**Tsx gA1**	AGGCAUUUAGGAUACUACAG
**Tsx gA2**	UCAAGGAGCACAGAUGGUGG
**Tsx gA3**	GAACCAGCGCCAUGACAGGU
**Tsx gA4**	UGGGGUGGGGUCAAGUUGAG
**Alb gA1**	CCAGAUGGCAAACAUACGCA
**Alb gA2**	AGUCUUGUGCAUGGGGGUGG
**Alb gA3**	GUAGGAACCAAUGAAAUGCG
**Rs1 gA1**	UGAUGUCGGAGAAAGAAUUA
**Rs1 gA2**	GAAAGUAGAGUUGGAAGAUG
**Rs1 gA3**	ACUCGCCUUACAGUUAAAGA
**Celrr gA1**	AGUGGGUUGGGAUGUGGGGG
**Celrr gA2**	UUUCCAUAUGAAAGCAGCUG
**Celrr gA3**	AGGUUCACUGAACUUGGUGU
**Cd274 gA1**	UGGACAAGGCUUCCGCGGAG
**Cd274 gA2**	UGAGAUAAACUCAUGCUCAA
**Cd274 gA3**	UCUGAACUCGAGAUAAGACC
**Tmem97 gA1**	GCUGCUGGGAGUACCGCGUG
**Tmem97 gA2**	CCCCGCUAUAUGGGCGGGCC
**Tmem97 gA3**	AGACCAGCGCACGCAGCCCG

Supplementary Table 15: Catalog numbers for ThermoFisher Scientific gene expression assays used for RT-qPCR in these studies.

Target Gene	Assay ID from ThermoFisher Scientific
Ttr	Mm00443267_m1
Ldlr	Mm01177349_m1
Pcsk9	Mm01263610_m1
Tsx	Mm00493902_m1
C9orf72	Mm01216829_m1
Rs1	Mm00488076_m1
Alb	Mm00802090_m1
Chic1	Mm01232479_m1
Tmem97	Mm01608791_g1
Celrr	Mm01309264_m1
Xist	Mm01232884_m1
Ppef1	Mm01156845_m1
Cdkl5	Mm01156815_m1
Ankrd17	Mm01344843_m1
Afp	Mm00431715_m1
Dsg2	Mm00514608_m1
B4Galt6	Mm00480045_m1

Supplementary Table 16: Custom gene expression assays designed to support these studies.

Designed Assay:	Forward Seq	Reverse Seq	Probe
dCas9-SAM	CCGACGCTAATCTGGACAAAGTG	GTCAGGGTAAACAGGTGGATGA	CTGTCCGCCTACAACAAGCACCG
B2m	GGGAAGCCGAACATACTGAACTG	CCCGTTCTTCAGCATTTGGATTTC	ACGTAACACAGTTCCACCCGCCT
Ngn3	CCCGGATGACGCCAAACTTAC	TCAGTGCCCAGATGTAGTTGTG	AAAGATCGAGACCCTGCGCTTCGCC
Btc	GCAGAGGCAGGCAAATCTC	GACAGTGTTCCTCTGTTGTAGTCTT	TGAGTTCAAGGCCAGCCTGGTCT
Ppef1	AGCTCCGTGACCACAGTAGG	TCAATGGTTACCAGGTGTGAAC	AAGGATTTCATTGGCCGAGTGGGCA
Cdkl5	GGAGTGGCGACCTGAGAAG	GAAGAAGCTGGATGATTGGTTGATG	TCAGATCTACAGACCCAGAGCCAACCA

Supplementary Table 17: Nucleotide sequences utilized in this study

dCas9SAM	ATGAAAAGGCCGGCGGCCACGAAAAAGGCCGGCCAGGCAAAAAAGAAAAAGGACAAGAAGTACAGCATCGGCC
	TGGCCATCGGCACCAACTCTGTGGGCTGGGCCGTGATCACCGACGAGTACAAGGTGCCCAGCAAGAAATTCAA
	GGTGCTGGGCAACACCGACCGGCACAGCATCAAGAAGAACCTGATCGGAGCCCTGCTGTTCGACAGCGGCGAA
	ACAGCCGAGGCCACCCGGCTGAAGAGAACCGCCAGAAGAAGATACACCAGACGGAAGAACCGGATCTGCTATC
	TGCAAGAGATCTTCAGCAACGAGATGGCCAAGGTGGACGACAGCTTCTTCCACAGACTGGAAGAGTCCTTCCTG
	GTGGAAGAGGATAAGAAGCACGAGCGGCACCCCATCTTCGGCAACATCGTGGACGAGGTGGCCTACCACGAGA
	AGTACCCCACCATCTACCACCTGAGAAAGAAACTGGTGGACAGCACCGACAAGGCCGACCTGCGGCTGATCTAT
	CTGGCCCTGGCCCACATGATCAAGTTCCGGGGCCACTTCCTGATCGAGGGCGACCTGAACCCCGACAACAGCG
	ACGTGGACAAGCTGTTCATCCAGCTGGTGCAGACCTACAACCAGCTGTTCGAGGAAAACCCCATCAACGCCAGC
	GGCGTGGACGCCAAGGCCATCCTGTCTGCCAGACTGAGCAAGAGCAGACGGCTGGAAAATCTGATCGCCCAGC
	TGCCCGGCGAGAAGAAGAATGGCCTGTTCGGCAACCTGATTGCCCTGAGCCTGGGCCTGACCCCCAACTTCAA
	GAGCAACTTCGACCTGGCCGAGGATGCCAAACTGCAGCTGAGCAAGGACACCTACGACGACGACCTGGACAAC
	CTGCTGGCCCAGATCGGCGACCAGTACGCCGACCTGTTTCTGGCCGCCAAGAACCTGTCCGACGCCATCCTGC
	TGAGCGACATCCTGAGAGTGAACACCGAGATCACCAAGGCCCCCCTGAGCGCCTCTATGATCAAGAGATACGA
	CGAGCACCACCAGGACCTGACCCTGCTGAAAGCTCTCGTGCGGCAGCAGCTGCCTGAGAAGTACAAAGAGATT
	TTCTTCGACCAGAGCAAGAACGGCTACGCCGGCTACATTGACGGCGGAGCCAGCCAGGAAGAGTTCTACAAGT
	TCATCAAGCCCATCCTGGAAAAGATGGACGGCACCGAGGAACTGCTCGTGAAGCTGAACAGAGAGGACCTGCT
	GCGGAAGCAGCGGACCTTCGACAACGGCAGCATCCCCCACCAGATCCACCTGGGAGAGCTGCACGCCATTCTG
	CGGCGGCAGGAAGATTTTTACCCATTCCTGAAGGACAACCGGGAAAAGATCGAGAAGATCCTGACCTTCCGCAT
	CCCCTACTACGTGGGCCCTCTGGCCAGGGGAAACAGCAGATTCGCCTGGATGACCAGAAAGAGCGAGGAAACC
	ATCACCCCCTGGAACTTCGAGGAAGTGGTGGACAAGGGCGCTTCCGCCCAGAGCTTCATCGAGCGGATGACCA
	ACTTCGATAAGAACCTGCCCAACGAGAAGGTGCTGCCCAAGCACAGCCTGCTGTACGAGTACTTCACCGTGTAT
	AACGAGCTGACCAAAGTGAAATACGTGACCGAGGGAATGAGAAAGCCCGCCTTCCTGAGCGGCGAGCAGAAAA
	AGGCCATCGTGGACCTGCTGTTCAAGACCAACCGGAAAGTGACCGTGAAGCAGCTGAAAGAGGACTACTTCAA
	GAAAATCGAGTGCTTCGACTCCGTGGAAATCTCCGGCGTGGAAGATCGGTTCAACGCCTCCCTGGGCACATACC
	ACGATCTGCTGAAAATTATCAAGGACAAGGACTTCCTGGACAATGAGGAAAACGAGGACATTCTGGAAGATATC
	GTGCTGACCCTGACACTGTTTGAGGACAGAGAGATGATCGAGGAACGGCTGAAAACCTATGCCCACCTGTTCGA
	CGACAAAGTGATGAAGCAGCTGAAGCGGCGGAGATACACCGGCTGGGGCAGGCTGAGCCGGAAGCTGATCAA
	CGGCATCCGGGACAAGCAGTCCGGCAAGACAATCCTGGATTTCCTGAAGTCCGACGGCTTCGCCAACAGAAAC
	TTCATGCAGCTGATCCACGACGACAGCCTGACCTTTAAAGAGGACATCCAGAAAGCCCAGGTGTCCGGCCAGG
	GCGATAGCCTGCACGAGCACATTGCCAATCTGGCCGGCAGCCCCGCCATTAAGAAGGGCATCCTGCAGACAGT
	GAAGGTGGTGGACGAGCTCGTGAAAGTGATGGGCCGGCACAAGCCCGAGAACATCGTGATCGAAATGGCCAGA
	GAGAACCAGACCACCCAGAAGGGACAGAAGAACAGCCGCGAGAGAATGAAGCGGATCGAAGAGGGCATCAAA
	GAGCTGGGCAGCCAGATCCTGAAAGAACACCCCGTGGAAAACACCCAGCTGCAGAACGAGAAGCTGTACCTGT
	ACTACCTGCAGAATGGGCGGGATATGTACGTGGACCAGGAACTGGACATCAACCGGCTGTCCGACTACGATGT
	GGACCACATCGTGCCTCAGAGCTTTCTGAAGGACGACTCCATCGACAACAAGGTGCTGACCAGAAGCGACAAG
	GCCCGGGGCAAGAGCGACAACGTGCCCTCCGAAGAGGTCGTGAAGAAGATGAAGAACTACTGGCGGCAGCTG
	CTGAACGCCAAGCTGATTACCCAGAGAAAGTTCGACAATCTGACCAAGGCCGAGAGAGGCGGCCTGAGCGAAC
	TGGATAAGGCCGGCTTCATCAAGAGACAGCTGGTGGAAACCCGGCAGATCACAAAGCACGTGGCACAGATCCT
	GGACTCCCGGATGAACACTAAGTACGACGAGAATGACAAGCTGATCCGGGAAGTGAAAGTGATCACCCTGAAGT
	CCAAGCTGGTGTCCGATTTCCGGAAGGATTTCCAGTTTTACAAAGTGCGCGAGATCAACAACTACCACCACGCC
	CACGACGCCTACCTGAACGCCGTCGTGGGAACCGCCCTGATCAAAAAGTACCCTAAGCTGGAAAGCGAGTTCG
	TGTACGGCGACTACAAGGTGTACGACGTGCGGAAGATGATCGCCAAGAGCGAGCAGGAAATCGGCAAGGCTAC
	CGCCAAGTACTTCTTCTACAGCAACATCATGAACTTTTTCAAGACCGAGATTACCCTGGCCAACGGCGAGATCCG
	GAAGCGGCCTCTGATCGAGACAAACGGCGAAACCGGGGAGATCGTGTGGGATAAGGGCCGGGATTTTGCCAC
	CGTGCGGAAAGTGCTGAGCATGCCCCAAGTGAATATCGTGAAAAAGACCGAGGTGCAGACAGGCGGCTTCAGC
	AAAGAGTCTATCCTGCCCAAGAGGAACAGCGATAAGCTGATCGCCAGAAAGAAGGACTGGGACCCTAAGAAGTA
	CGGCGGCTTCGACAGCCCCACCGTGGCCTATTCTGTGCTGGTGGTGGCCAAAGTGGAAAAGGGCAAGTCCAAG
	AAACTGAAGAGTGTGAAAGAGCTGCTGGGGATCACCATCATGGAAAGAAGCAGCTTCGAGAAGAATCCCATCGA
	CTTTCTGGAAGCCAAGGGCTACAAAGAAGTGAAAAAGGACCTGATCATCAAGCTGCCTAAGTACTCCCTGTTCG
	AGCTGGAAAACGGCCGGAAGAGAATGCTGGCCTCTGCCGGCGAACTGCAGAAGGGAAACGAACTGGCCCTGC
	CCTCCAAATATGTGAACTTCCTGTACCTGGCCAGCCACTATGAGAAGCTGAAGGGCTCCCCCGAGGATAATGAG
	CAGAAACAGCTGTTTGTGGAACAGCACAAGCACTACCTGGACGAGATCATCGAGCAGATCAGCGAGTTCTCCAA
	GAGAGTGATCCTGGCCGACGCTAATCTGGACAAAGTGCTGTCCGCCTACAACAAGCACCGGGATAAGCCCATC
	AGAGAGCAGGCCGAGAATATCATCCACCTGTTTACCCTGACCAATCTGGGAGCCCCTGCCGCCTTCAAGTACTT
	TGACACCACCATCGACCGGAAGAGGTACACCAGCACCAAAGAGGTGCTGGACGCCACCCTGATCCACCAGAGC
	ATCACCGGCCTGTACGAGACACGGATCGACCTGTCTCAGCTGGGAGGCGAC
VP64	GCGGCCGCTGGATCCGGACGGGCTGACGCATTGGACGATTTTGATCTGGATATGCTGGGAAGTGACGCCCTCGATG
	ATTTTGACCTTGACATGCTTGGTTCGGATGCCCTTGATGACTTTGACCTCGACATGCTCGGCAGTGACGCCCTTGATG
	ATTTCGACCTGGACATGCTGATTAACTGTACAG

MCP	ATGGCTTCAAACTTTACTCAGTTCGTGCTCGTGGACAATGGTGGGACAGGGGATGTGACAGTG<br>GCTCCTTCTAATTTCGCTAATGGGGTGGCAGAGTGGATCAGCTCCAACTCACGGAGCCAGGCCTACAAGGTGAC<br>ATGCAGCGTCAGGCAGTCTAGTGCCCAGAAGAGAAAGTATACCATCAAGGTGGAGGTCCCCAAAGTGGCTACC<br>CAGACAGTGGGCGGAGTCGAACTGCCTGTCGCCGCTTGGAGGTCCTACCTGAACATGGAGCTCACTATCCCAA<br>TTTTCGCTACCAATTCTGACTGTGAACTCATCGTGAAGGCAATGCAGGGGCTCCTCAAAGACGGTAATCCTATCC<br>CTTCCGCCATCGCCGCTAACTCAGGTATCTAC
P65	CCTTCAGGGCAGATCAGCAACCAGGCCCTGGCTCTGGCCCCTAGCTCCGCTCCAGTGCTGGCCCAGACTATGGTG<br>CCCTCTAGTGCTATGGTGCCTCTGGCCCAGCCACCTGCTCCAGCCCCTGTGCTGACCCCAGGACCACCCCAGTCA<br>CTGAGCGCTCCAGTGCCCAAGTCTACACAGGCCGGCGAGGGGACTCTGAGTGAAGCTCTGCTGCACCTGCAGTTCG<br>ACGCTGATGAGGACCTGGGAGCTCTGCTGGGGAACAGCACCGATCCCGGAGTGTTCACAGATCTGGCCTCCGTGGA<br>CAACTCTGAGTTTCAGCAGCTGCTGAATCAGGGCGTGTCCATGTCTCATAGTACAGCCGAACCAATGCTGATGGAGT<br>ACCCCGAAGCCATTACCCGGCTGGTGACCGGCAGCCAGCGGCCCCCCGACCCCGCTCCAACTCCCCTGGGAACC<br>AGCGGCCTGCCTAATGGGCTGTCCGGAGATGAAGACTTCTCAAGCATCGCTGATATGGACTTTAGTGCCCTGCTGTC<br>ACAGATTTCCTCT
HSF1	GGCTTCAGCGTGGACACCAGTGCCCTGCTGGACCTGTTCAGCCCCTCGGTGACCGTGCCCGACATGAGCCTGCCT<br>GACCTTGACAGCAGCCTGGCCAGTATCCAAGAGCTCCTGTCTCCCCAGGAGCCCCCCAGGCCTCCCGAGGCAGAG<br>AACAGCAGCCCGGATTCAGGGAAGCAGCTGGTGCACTACACAGCGCAGCCGCTGTTCCTGCTGGACCCCGGCTCC<br>GTGGACACCGGGAGCAACGACCTGCCGGTGCTGTTTGAGCTGGGAGAGGGCTCCTACTTCTCCGAAGGGGACGGC<br>TTCGCCGAGGACCCCACCATCTCCCTGCTGACAGGCTCGGAGCCTCCCAAAGCCAAGGACCCCACTGTCTCCTGA
hU6	TTTCCCATGATTCCTTCATATTTGCATATACGATACAAGGCTGTTAGAGAGATAATTGGAATTAATTTGACTGTAAA<br>CACAAAGATATTAGTACAAAATACGTGACGTAGAAAGTAATAATTTCTTGGGTAGTTTGCAGTTTTAAAATTATGTT<br>TTAAAATGGACTATCATATGCTTACCGTAACTTGAAAGTATTTCGATTTCTTGGCTTTATATATCTTGTGGAAAGGA<br>CGAAACACC
SAM Tracr	GTTTTAGAGCTAGGCCAACATGAGGATCACCCATGTCTGCAGGG<br>CCTAGCAAGTTAAAATAAGGCTAGTCCGTTATCAACTTGGCCAACATGAGGATCACCCATGTCTGCAGGGCCAA<br>GTGGCACCGAGTCGGTGCT
Extended Pol III termination sequence	TTTTTTGTTTTAGAGCTAGAAATAGCAAGTTAAAATAAGGCTAGTCCGTTTT

Supplementary Table 18: dCas9 amino acid sequence

dCas9SAM	MKRPAATKKAGQAKKKKDKKYSIGLAIGTNSVGWAVITDEYKVPSKKFKVLGNTDRHSIKKNLIGALLFDSGETAEATR<br>LKRTARRRYTRRKNRICYLQEIFSNEMAKVDDSFFHRLEESFLVEEDKKHERHPIFGNIVDEVAYHEKYPTIYHLRKKLV<br>DSTDKADLRLIYLALAHMIKFRGHFLIEGDLNPDNSDVDKLFIQLVQTYNQLFEENPINASGVDAKAILSARLSKSRRLE<br>NLIAQLPGEKKNGLFGNLIALSLGLTPNFKSNFDLAEDAKLQLSKDTYDDDLDNLLAQIGDQYADLFLAAKNLSDAILLS<br>DILRVNTEITKAPLSASMIKRYDEHHQDLTLLKALVRQQLPEKYKEIFFDQSKNGYAGYIDGGASQEEFYKFIKPILEKM<br>DGTEELLVKLNREDLLRKQRTFDNGSIPHQIHLGELHAILRRQEDFYPFLKDNREKIEKILTFRIPYYVGPLARGNSRFA<br>WMTRKSEETITPWNFEEVVDKGASAQSFIERMTNFDKNLPNEKVLPKHSLLYEYFTVYNELTKVKYVTEGMRKPAFLS<br>GEQKKAIVDLLFKTNRKVTVKQLKEDYFKKIECFDSVEISGVEDRFNASLGTYHDLLKIIKDKDFLDNEENEDILEDIVLTL<br>TLFEDREMIEERLKTYAHLFDDKVMKQLKRRRYTGWGRLSRKLINGIRDKQSGKTILDFLKSDGFANRNFMQLIHDDS<br>LTFKEDIQKAQVSGQGDSLHEHIANLAGSPAIKKGILQTVKVVDELVKVMGRHKPENIVIEMARENQTTQKGQKNSRE<br>RMKRIEEGIKELGSQILKEHPVENTQLQNEKLYLYYLQNGRDMYVDQELDINRLSDYDVDHIVPQSFLKDDSIDNKVLT<br>RSDKARGKSDNVPSEEVVKKMKNYWRQLLNAKLITQRKFDNLTKAERGGLSELDKAGFIKRQLVETRQITKHVAQILD<br>SRMNTKYDENDKLIREVKVITLKSKLVSDFRKDFQFYKVREINNYHHAHDAYLNAVVGTALIKKYPKLESEFVYGDYKV<br>YDVRKMIAKSEQEIGKATAKYFFYSNIMNFFKTEITLANGEIRKRPLIETNGETGEIVWDKGRDFATVRKVLSMPQVNIV<br>KKTEVQTGGFSKESILPKRNSDKLIARKKDWDPKKYGGFDSPTVAYSVLVVAKVEKGKSKKLKSVKELLGITIMERSSF<br>EKNPIDFLEAKGYKEVKKDLIIKLPKYSLFELENGRKRMLASAGELQKGNELALPSKYVNFLYLASHYEKLKGSPEDNE<br>QKQLFVEQHKHYLDEIIEQISEFSKRVILADANLDKVLSAYNKHRDKPIREQAENIIHLFTLTNLGAPAAFKYFDTTIDRKR<br>YTSTKEVLDATLIHQSITGLYETRIDLSQLGGDSAGGGGSGGGGSGGGGSGPKKKRKVAAAGSGRADALDDFDLDM<br>LGSDALDDFDLDMLGSDALDDFDLDMLGSDALDDFDLDMLINCTGSGEGRGSLLTCGDVEENPGPMASNFTQFVLV<br>DNGGTGDVTVAPSNFANGVAEWISSNSRSQAYKVTCSVRQSSAQKRKYTIKVEVPKVATQTVGGVELPVAAWRSYL<br>NMELTIPIFATNSDCELIVKAMQGLLKDGNPIPSAIAANSGIYSAGGGGSGGGGSGGGGSGPKKKRKVAAAGSPSGQI<br>SNQALALAPSSAPVLAQTMVPSSAMVPLAQPPAPAPVLTPGPPQSLSAPVPKSTQAGEGTLSEALLHLQFDADEDLG<br>ALLGNSTDPGVFTDLASVDNSEFQQLLNQGVSMSHSTAEPMLMEYPEAITRLVTGSQRPPDPAPTPLGTSGLPNGLS<br>GDEDFSSIADMDFSALLSQISSSGQGGGGSGFSVDTSALLDLFSPSVTVPDMSLPDLDSSLASIQELLSPQEPPRPPE<br>AENSSPDSGKQLVHYTAQPLFLLDPGSVDTGSNDLPVLFELGEGSYFSEGDGFAEDPTISLLTGSEPPKAKDPTVS
