# Supplementary material for: Tissue-specific activation of gene expression by the Synergistic Activation Mediator (SAM) CRISPRa system in mice
Source: Nat Commun. 2021 May 13;12:2770. doi: 10.1038/s41467-021-22932-4 (PMC8119962; doi:10.1038/s41467-021-22932-4)
Supplement: Supplementary file 3 — Reporting Summary [file 41467_2021_22932_MOESM3_ESM.pdf]

nature research

Corresponding author(s): Guochun Gong

Last updated by author(s): 2021/01/04

Reporting Summary

Nature Research wishes to improve the reproducibility of the work that we publish. This form provides structure for consistency and transparency in reporting. For further information on Nature Research policies, see our Editorial Policies and the Editorial Policy Checklist.

Statistics

For all statistical analyses, confirm that the following items are present in the figure legend, table legend, main text, or Methods section.

n/a	Confirmed	
☐	☒	The exact sample size (*n*) for each experimental group/condition, given as a discrete number and unit of measurement
☒	☐	A statement on whether measurements were taken from distinct samples or whether the same sample was measured repeatedly
☐	☒	The statistical test(s) used AND whether they are one- or two-sided<br>*Only common tests should be described solely by name; describe more complex techniques in the Methods section.*
☒	☐	A description of all covariates tested
☒	☐	A description of any assumptions or corrections, such as tests of normality and adjustment for multiple comparisons
☐	☒	A full description of the statistical parameters including central tendency (e.g. means) or other basic estimates (e.g. regression coefficient) AND variation (e.g. standard deviation) or associated estimates of uncertainty (e.g. confidence intervals)
☐	☒	For null hypothesis testing, the test statistic (e.g. *F*, *t*, *r*) with confidence intervals, effect sizes, degrees of freedom and *P* value noted<br>*Give P values as exact values whenever suitable.*
☒	☐	For Bayesian analysis, information on the choice of priors and Markov chain Monte Carlo settings
☒	☐	For hierarchical and complex designs, identification of the appropriate level for tests and full reporting of outcomes
☐	☒	Estimates of effect sizes (e.g. Cohen's *d*, Pearson's *r*), indicating how they were calculated

Our web collection on statistics for biologists contains articles on many of the points above.

Software and code

Policy information about availability of computer code

Data collection	ADVIA Chemistry XPT system (tailored to mouse levels)<br>20x objective Hamamazu camera (AxioScan.Z1)<br>Halo version 3.0.311.398<br>ArrayStudio OmicSoft® version 10<br>DESeq2 version 1.10.1<br>FastQC version 0.11.5<br>Adobe Photoshop CC version 19.1.5<br>GraphPad Prism version 8.2.0<br>MacVector with Assembler version 17.5.2
Data analysis	Raw RNA sequence data (BCL files) were converted to FASTQ format via Illumina bcl2fastq v2.17. Reads were decoded based on their barcodes and read quality was evaluated with FastQC (http://www.bioinformatics.babraham.ac.uk/projects/fastqc/). Reads were mapped to the mouse genome (NCBI GRCm38) using ArrayStudio® software (OmicSoft®, Cary, NC) allowing two mismatches. Reads mapped to the exons of a gene were summed at the gene level. Genes were flagged as detectable with minimum 10 reads. Differentially expressed genes were identified by DESeq2 package

For manuscripts utilizing custom algorithms or software that are central to the research but not yet described in published literature, software must be made available to editors and reviewers. We strongly encourage code deposition in a community repository (e.g. GitHub). See the Nature Research guidelines for submitting code & software for further information.

Data

Policy information about availability of data

All manuscripts must include a data availability statement. This statement should provide the following information, where applicable:

- Accession codes, unique identifiers, or web links for publicly available datasets
- A list of figures that have associated raw data
- A description of any restrictions on data availability

Competing Interests: CHu, SH, DW, EP, TH, CHe, JW, HB, QS, DV, JA, KC, JH, SC, MD, SM-T, MPM-P, GD, DF, EC, BZ, and GG are employees of Regeneron Pharmaceuticals Inc ("Regeneron"). Regeneron has filed patent applications around the described work. The remaining authors declare no competing interests.

Data and materials availability:
Source data associated with this study are present in the paper or the Supplementary Materials. Raw RNAseq data can be accessed from BioProject ID PRJNA669145 (https://www.ncbi.nlm.nih.gov/bioproject/PRJNA669145). Materials described in this manuscript are available by contacting Regeneron Pharmaceuticals, Inc. (email address: preclinical.collaborations@regeneron.com) for academic and non-profit purposes only under an MTA, which allows the use of mice for academic but not commercial purposes.

Field-specific reporting

Please select the one below that is the best fit for your research. If you are not sure, read the appropriate sections before making your selection.

☒ Life sciences ☐ Behavioural & social sciences ☐ Ecological, evolutionary & environmental sciences

For a reference copy of the document with all sections, see nature.com/documents/nr-reporting-summary-flat.pdf

Life sciences study design

All studies must disclose on these points even when the disclosure is negative.

Sample size	Sample sizes were determined based on expected effect sizes and technical limitations. The significance of the results obtained suggests that the chosen sample sizes are appropriate
Data exclusions	No data was excluded
Replication	2 repeat experiments were conducted for lipid modulation and all yielded similar results.
Randomization	Randomization was not performed because groups were defined by genotype.
Blinding	Blinding was not considered necessary as the data analysts lacked prior knowledge of the underlying biological process that could have biased their expectations

Reporting for specific materials, systems and methods

We require information from authors about some types of materials, experimental systems and methods used in many studies. Here, indicate whether each material, system or method listed is relevant to your study. If you are not sure if a list item applies to your research, read the appropriate section before selecting a response.

Materials & experimental systems

n/a	Involved in the study
☐	☒ Antibodies
☐	☒ Eukaryotic cell lines
☒	☐ Palaeontology and archaeology
☐	☒ Animals and other organisms
☒	☐ Human research participants
☒	☐ Clinical data
☒	☐ Dual use research of concern

Methods

n/a	Involved in the study
☒	☐ ChIP-seq
☒	☐ Flow cytometry
☒	☐ MRI-based neuroimaging

Antibodies

Antibodies used	AntiCas9: Thermo MA1201<br>HRP-conjugated anti-beta-actin: Millipore Sigma MAB1501
Validation	AntiCas9 was manufacturer-validated on U2OS cells with stable Cas9 expression

AntiActin was manufacturer-validated on HeLa whole cell lysates

Eukaryotic cell lines

Policy information about cell lines

Cell line source(s)	Mouse ES cells were derived in house from B6/129 hybrid mice and referred to as WT cells in the text
Authentication	This cell line was not authenticated by any commercial service. Cells have gone through extensive karyotype screening as well as expression characterization.
Mycoplasma contamination	These cells test negative for mycoplasm.
Commonly misidentified lines (See ICLAC register)	No commonly misidentified cell lines were used in the study

Animals and other organisms

Policy information about studies involving animals; ARRIVE guidelines recommended for reporting animal research

Laboratory animals	B6/129 hybrid mice were used in all studies. Mice were housed at ambient temperature (68-74F) on a 12 hour light/dark cycle with 40-50% humidity. Male and female mice were utilized between 10-15 weeks, depending on the study.
Wild animals	No wild animals were utilized
Field-collected samples	Samples were not collected in the field
Ethics oversight	Regeneron Institutional Animal Care and Use Committee (IACUC)

Note that full information on the approval of the study protocol must also be provided in the manuscript.
